# 6S-1 pRNA 9-mers are a prominent length species during outgrowth of *Bacillus subtilis* cells from extended stationary phase

**DOI:** 10.1080/15476286.2025.2484519

**Published:** 2025-03-25

**Authors:** Katrin Damm, Paul Klemm, Marcus Lechner, Dominik Helmecke, Roland K. Hartmann

**Affiliations:** a Fachbereich Pharmazie, Institut für Pharmazeutische Chemie, Philipps-Universität Marburg, Marburg, Germany; b Center for Synthetic Microbiology (SYNMIKRO), Philipps-Universität Marburg, Marburg, Germany

**Keywords:** RNA-Seq, Illumina sequencing, polyA/C/U-tailing, polyA/U polymerase, pRNAs, *Bacillus subtilis* 6S-1 RNA

## Abstract

Bacterial RNA polymerases (RNAP) utilize 6S RNAs as templates to synthesize ultrashort transcripts (up to ~14 nt), termed product RNAs (pRNAs), that play a key role in reversing the blockage of RNAP by 6S RNA. Here, we resolved the pRNA length profile of 6S-1 RNA from *B. subtilis*, a major model system for the study of 6S RNA biology, during outgrowth of cells from extended stationary phase. 9-mers were found to be a particularly abundant pRNA length species, followed by 8-/10-/11-mers and 13-/14-mers. Consistent with *in vitro* data from the *Escherichia coli* system, these findings support the mechanistic model according to which the housekeeping sigma factor (σ^70^ or σ^A^) dissociates from 6S RNA:RNAP complexes upon synthesis of pRNA 9-mers, followed by final dissociation of 6S RNA and RNAP upon synthesis of longer pRNAs (13-/14-mers). Methodologically, the identification of such ultrashort RNAs in total cellular extracts by RNA-Seq is inefficient with standard protocols using adapter ligation to RNA 3’-ends for reverse transcription and PCR-based cDNA sequencing. Here, we demonstrate that ultrashort RNAs can instead be incorporated into RNA-Seq libraries by polyA-, polyC- and potentially also polyU-tailing of their 3’-ends. At positions where a non-tailing nucleotide is followed by one or more tailing nucleotides, an algorithm that integrates RNA-Seq results from at least two different 3’-end tailings allows one to approximate the fraction of read counts at such ambiguous positions. Finally, methodological biases and potential applications of our approach to other short RNAs are discussed.

## Introduction

The majority of bacteria encode a single non-coding 6S RNA [1] that binds to the RNA polymerase (RNAP) holoenzyme containing the primary housekeeping sigma factor (σ^70^ in *Escherichia coli*, σ^A^ in *Bacillus subtilis*). Some bacteria including *B. subtilis* encode two 6S RNA paralogs (6S–1 and 6S–2 RNA) [1]. 6S RNAs, ~200 nt in length, adopt a rod-shaped secondary structure consisting of imperfectly paired helical arms separated by a less structured region in their centre (named central bulge or central bubble). This overall RNA structure mimics an open DNA promoter and competes with DNA promoters for binding to RNAP holoenzymes [2]. Bacterial strains with 6S RNA gene deletions showed transcriptomic and proteomic dysregulations particularly under various stress conditions [3–7]. As a result of the open DNA promoter mimicry, σ^A/70^-RNAPs can utilize the RNA as a template for the transcription of short transcripts, termed product RNAs (pRNAs) [8]. For pRNA synthesis on *B. subtilis* 6S–1 RNA as template, the pRNA amount and length pattern was found to increase with rising NTP concentration, especially of the initiating nucleotide (GTP in the case of 6S–1 pRNAs) [8–11]. With increasing pRNA length, the pRNA dissociation rate from 6S RNA decreases, reaching dwell times during which the 6S RNA:pRNA complex with a substantially refolded 6S RNA structure becomes long-lasting enough to break the interactions with RNAP. This then allows the enzyme to escape from the transcriptional block imposed by 6S RNA [8,10,12]. This release mechanism is operational when bacterial cells leave phases of growth stagnation upon resupply of nutrients, which is associated with rising NTP levels [5,10].

Based on *in vitro* studies, pRNAs in the *E. coli* system were reported to be 14 to 24 nt in length [8,13]. Subsequent in-depth *in vitro* analyses in the *E. coli* system revealed two pRNA length variants to play a key role in the functional cycle of 6S RNA: pRNA 9-mers triggered the release of σ^70^ from RNAP and pRNA extension to 13-mers then resulted in 6S RNA dissociation from the core RNAP [14].

In the case of *B. subtilis* 6S–1 RNA, we previously performed RNA-Seq employing 3’-polyA-tailing of small RNAs for library construction [9]. In outgrowth conditions, the study identified primarily reads corresponding to pRNA 8- to 12-mers and 14-/15-mers. Owing to the 6S–1 pRNA sequence (5’-GUUCGGUCAAAACUA), the polyA-tailing approach failed to resolve the length distribution between nt 8 to 12 and 14/15. In view of the findings in the *E. coli* system, where pRNA 9- and 13-mers were identified as key length species in the release mechanism, we additionally explored 3’-polyC- and polyU-tailing of small RNAs as well as 3’-adapter ligation for RNA-Seq library construction, aiming at fully resolving the 6S–1 pRNA length pattern during outgrowth of *B. subtilis* cells from extended stationary phase.

As very short RNAs have a low information content, they are poorly amenable to Northern blot detection. Thus, RNA-Seq approaches are required to identify their authentic length spectra within cells, based on libraries with quantitatively significant numbers of reads. We, therefore, evaluate our methodology also in regard to its applicability to other types of short RNAs.

## Results

### *In vitro* polyC-tailing of RNA 8- and 14-mers mimicking 6S–1 pRNAs

**Figure 1.**
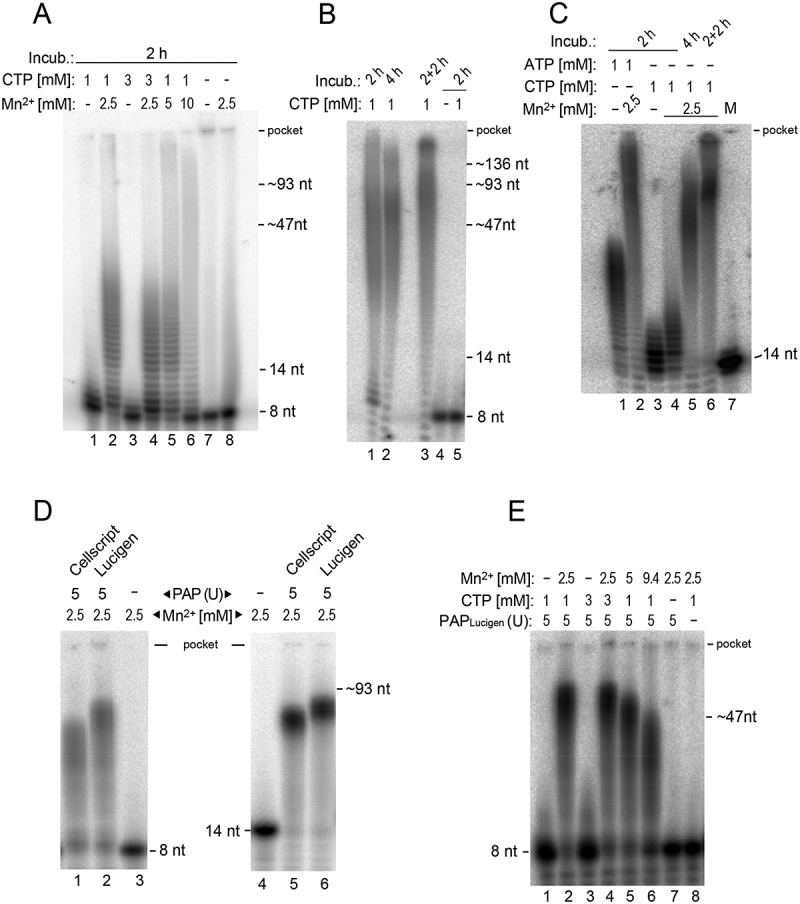
Variation of conditions for polyC-tailing of an RNA 8- or 14-mer. (A) In lane 1, the synthetic 6S–1 pRNA 8-mer (5’-GUU CGG UC-3’; 1 µm) containing trace amounts of the sequence-identical 5’-^32^P-labelled pRNA (10,000 Cherenkov cpm) was incubated for 2 h at 37°C with 1 mM CTP, 4 mM DTT and 5 units of polyA polymerase (NEB) in 1× reaction buffer (50 mM Tris-HCl, 250 mM NaCl, 10 mM MgCl_2_, ~pH 8.0); the other lanes differed either by the CTP concentration (3 mM in lanes 3 and 4; no CTP in lanes 7 and 8) or by addition of MnCl_2_ at the indicated concentrations. (B) Variation of incubation under the same conditions as in panel A, but additionally containing 2.5 mM MnCl_2_; CTP was omitted in lane 4 and polyA polymerase in lane 5; 2 + 2 h: after 2 h at 37°C, another 5 units of polyA polymerase were added followed by incubation for another 2 h at 37°C. (C) Comparison of polyA- and polyC-tailing for the synthetic 6S–1 pRNA 14-mer (5’-GUU CGG UCA AAA CU-3’). The reaction setup was as in panel A, but using 1 mM ATP in lanes 1 and 2; 2.5 mM MnCl_2_ was added in lanes 2 and 4–6. Incubation conditions were: lanes 1–4, 2 h at 37°C; lane 5, 4 h at 37°C; lane 6, 2 h at 37°C, followed by addition of another 5 units of polyA polymerase and incubation for another 2 h at 37°C. M, the 5’-^32^P-labelled 14-mer used as length marker. (D) PolyC-tailing of the 8-mer and 14-mer, but using polyA polymerase (PAP) from Cellscript or Lucigene. Reactions conditions were as in panel A, lane 2, with enzyme omission controls in lanes 3 and 4; incubation was for 2 h at 37°C. (E) Variation of MnCl_2_ and CTP concentrations in polyC-tailing of the 8-mer by polyA polymerase from Lucigene. Otherwise, conditions were identical to panel D, lane 2; incubation was for 2 h at 37°C. All images show 20% PAA (acrylamide/bisacrylamide 24:1)/8 M urea gels. The gels in panels A-C were 24 cm long, 16 cm wide and 1 mm thick, those in panels D and E 20 cm long, 30 cm wide and 1 mm thick. The bottom of the gel pockets is indicated for each gel. Additional RNA size markers at the right margins of panels A, B, D and E were inferred from other 20% PAA/8 M urea gels of the same type, on which we separated RNAs of defined length together with an 8- or 14-mer. The swung dashes thus indicate that the positions of these size markers are approximations.

As a first step, we explored polyC-tailing for RNA-Seq of synthetic 6S–1 pRNA 8- and 14-mers. Assays with polyA polymerase and CTP as the nucleotide substrate revealed substantial C-tailing of a synthetic 6S–1 pRNA 8-mer (5’-GUU CGG UC-3’) in the presence of 2.5 mM Mn^2+^/10 mM Mg^2+^ (Figure 1(A), lanes 2 and 4). The majority of tails was in the range of ~7 to 20 C residues. Tailing efficiency was comparable at the higher Mn^2+^ concentration of 5 mM, whereas 10 mM Mn^2+^ led to reduced elongation (Figure 1(A), lanes 2 and 5 *vs*. 6). We also tested if higher CTP concentrations or longer incubation periods may stimulate polyC-tailing activity. Whereas the use of 3 instead of 1 mM CTP did not improve C-tailing efficiency (Figure 1(A), lane 4 *vs*. 2), a longer reaction time (4 instead of 2 h) somewhat increased overall efficiency (Figure 1(B), lane 2 *vs*. 1). The addition of fresh polyA polymerase at half time (2 h) further shifted the tail length distribution to longer variants (Figure 1(B), lane 3). Based on these results, we concluded that efficient polyC-tailing of RNA oligonucleotides as short as 8 nt is achieved in the presence of 2.5 mM Mn^2+^/10 mM Mg^2+^ and 1 mM CTP, applying the reaction protocol used in Figure 1(B), lane 3.

PolyC-tailing in comparison to polyA-tailing was addressed using the synthetic 6S–1 pRNA 14-mer (5’-GUU CGG UCA AAA CU-3’) (Figure 1(C)). In the presence of 2.5 mM Mn^2+^/10 mM Mg^2+^, polyA tails were longer than those obtained at 10 mM Mg^2+^ only (Figure 1(C), lane 2 *vs*. 1), indicating that the efficiency of polyA-tailing could be improved by addition of Mn^2+^ relative to standard protocols. A similar relation was observed for polyC-tailing in the absence versus presence of Mn^2+^ (Figure 1(C), lanes 3 and 4). As for the pRNA 8-mer, increasing the incubation time to 4 h, and particularly 4 h of incubation with addition of fresh enzyme after 2 h, resulted in polyC tails of sufficient length for stable annealing of oligo-(dG)_15_ primers (Figure 1(C), lanes 5 and 6 *vs*. 4).

We used polyA polymerase from NEB for the polyC-tailing step in the construction of small RNA libraries from *B. subtilis* (see below) under the conditions applied in Figure 1(B) (lane 3) and Figure 1(C) (lane 6). Nevertheless, we further tested polyA polymerases from two other suppliers at a later time point in the project to assess the efficiency of different commercially available polyA polymerases in polyC-tailing of very short RNAs. The two enzymes from Cellscript and Lucigene were efficient as well in polyC-tailing (Figure 1(D)) and, as shown for the enzyme from Lucigene, also required Mn^2+^ for robust polyC-tailing activity (Figure 1(E)).

### *In vitro* polyU-tailing of RNA 8- and 14-mers

**Figure 2.**
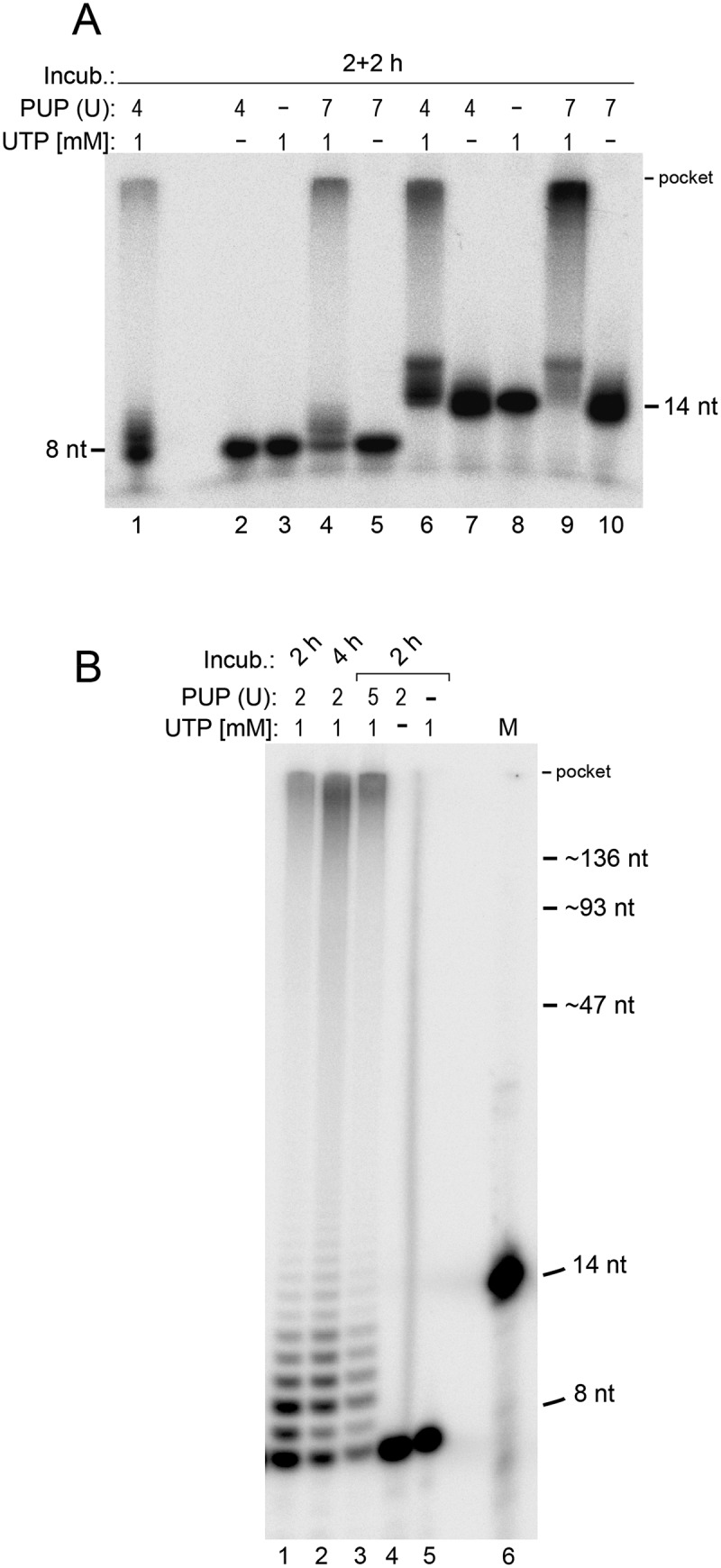
PolyU-tailing of the RNA 8- and 14-mer. (A) 1 µM RNA 8-mer (lanes 1–5) or 14-mer (lanes 6–10) containing trace amounts of the sequence-identical 5’-^32^P-labelled RNA (10,000 Cherenkov cpm) was incubated with polyU polymerase (PUP) in the presence of 1 mM UTP in 1× NEBuffer 2. In lanes 1, 2, 6 and 7, two U of enzyme were added and samples were incubated for 2 h at 37°C, followed by addition of another 2 U of enzyme and incubation for another 2 h at 37°C (4 U in sum). Lanes 4, 5, 9 and 10 differed in that 5 U of enzyme were added at the beginning of the reaction and another 2 U after the first 2 h of incubation (7 U in sum). UTP was omitted in lanes 2, 5, 7 and 10; polyU polymerase was omitted in lanes 3 and 8. (B) Extended electrophoresis runs to resolve products with shorter polyU tails. Incubation conditions, enzyme amounts and UTP concentration are indicated above the lanes. Otherwise, reactions were carried out as in panel A. M, 5’-^32^P-labelled 6S–1 pRNA 14-mer used as length marker. Gel dimensions in panel A were as in Figure 1(D). The gel in panel B was part of the same gel shown in Figure 1(B). For RNA size markers with swung dashes, see legend to Figure 1.

PolyU-tailing with a polyU polymerase (PUP) is another option beyond polyA- and polyC-tailing. Higher amounts of enzyme (added in two aliquots in the ‘2 + 2 h’ incubation format) resulted in substantial tailing, somewhat more efficient for the 14-mer than the 8-mer (Figure 2(A), lanes 4 and 9). With both oligomers, we observed two fractions of extension products, one with U tails of <10 residues, resolved in Figure 2(B) (lanes 1–3), and a second with very long tails migrating only at a short distance to the gel pocket (Figure 2(A), lanes 1, 4, 6 and 9; Figure 2(B), lanes 1–3); this distribution could be changed in favour of the long extension products by doubling the incubation time (Figure 2(B), lane 2) and by increasing the enzyme concentration (Figure 2(B), lane 3; Figure 2(A), cf. lanes 1 & 4 and 6 & 9). Addition of 2.5 mM Mn^2+^ did not improve U-tailing efficiency, neither for the 8-mer (Figure S1(A)) nor the 14-mer (Figure S1(C)). In conclusion, polyU-tailing particularly of the ultrashort RNA 8-mer by PUP tended to be less efficient than polyC-tailing. The molecules with U tails as short as 1 to 5 nt (Figure 2(B), lanes 1–3) are insufficient for binding of an oligo-dA primer. Thus, short RNA oligonucleotides are at risk to be underrepresented in corresponding RNA libraries that involve a polyU-tailing step. Multiple polyU-tailing reactions with phenol extraction and ethanol precipitation steps in between, combined with testing of other PUPs will likely help overcome this drawback.

### Northern blot analysis of short pRNAs

**Figure 3.**
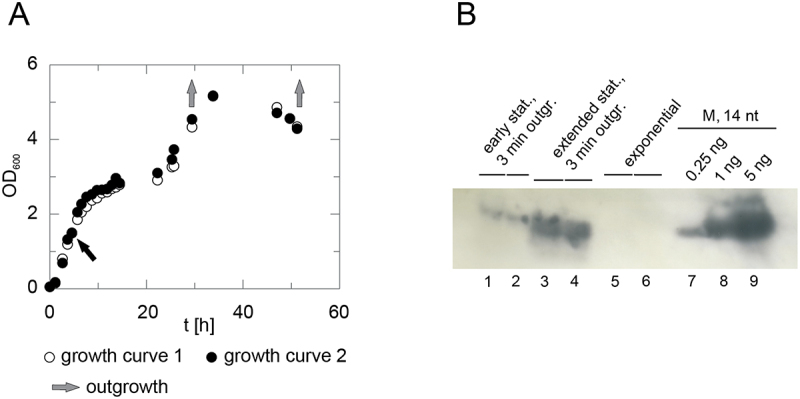
Analysis of 6S–1 pRNA expression in *B. subtilis*. (A) Two representative curves (open and filled circles) for the growth of *B. subtilis* PY79 in LB medium over a period of 50 h are shown. Grey arrows indicate time points in the growth curves (early and extended stationary phase) at which cells were diluted in fresh, prewarmed medium to induce outgrowth for 3 min, followed by cell harvest. The black arrow marks the time point at which cells in exponential phase were extracted. (B) Northern blot analysis of endogenous 6S–1 pRNAs in total cellular RNA (6 µg per lane) extracted at the different time points specified in panel A: either early stationary phase cells after 3 min of outgrowth (duplicate in lanes 1 and 2), extended stationary phase cells after 3 min of outgrowth (duplicate in lanes 3 and 4), or from cells harvested during exponential phase (duplicate in lanes 5 and 6). For more details, see Materials and methods. Note that the Northern blot detected mainly pRNA ~14-mers, but essentially no shorter pRNAs, for technical reasons (for more details, see [15] and [16]. Thus, pRNAs <14 nt are present in cellular RNA extracts but remain undetected in Northern blots. The chemically synthesized pRNA 14-mer (5’-GUU CGG UCA AAA CU-3’) was loaded as marker (M) in different amounts: 0.25 ng, 1 ng and 5 ng (lanes 7–9).

In preparation for the RNA-Seq analyses, cellular 6S–1 pRNA levels were examined by Northern blot experiments. For this purpose, *B. subtilis* cells were grown into extended stationary phase. At the onset of stationary phase, and in extended stationary phase when cell density already decreased, cells were diluted 1:5 in fresh prewarmed LB medium to induce outgrowth (Figure 3(A)). Cells were then harvested 3 min after induction of outgrowth and total RNAs were extracted for Northern blot analysis. In line with previous findings [9], strongest pRNA signals (~14 nt) were observed when outgrowth was initiated in extended stationary phase (Figure 3(B), lanes 3 and 4), was lower for outgrowth initiated at the onset of stationary phase (lanes 1 and 2), and was below the detection limit for cells harvested in exponential phase (OD_600_ of 1.5; lanes 5 and 6). We hence selected RNA samples outgrown from extended stationary phase for 3 min (Figure 3(B), lanes 3 and 4) for further RNA-Seq analysis. It should also be noted in this context that the applied Northern blot protocol did not detect pRNAs < 14 nt. We recently explored the detection of pRNA 8-mers using different protocols, demonstrating this to be a balancing act that yields at best sub-quantitative signals under low stringency conditions associated with ascending non-specific signals [16].

### Library construction for RNA-seq

After isolation of total RNA from *B. subtilis* PY79 cells, small RNA species <200 nt were enriched (see Materials and methods for details). Then equal RNA fractions were either subjected to polyadenylation of RNA 3’-ends, polycytidylation or 3’-adapter ligation. After PCR amplification, cDNAs representing RNAs ≤50 bp were size-fractionated on preparative 6% polyacrylamide gels, gel-eluted and subjected to Illumina sequencing. We further analysed a second library based on polyC-tailing, where we harvested the cells and enriched for small RNA species <200 nt as above, yet followed by further size fractionation by denaturing 15% PAGE to enrich for RNAs ≤50 nt already at this point. We obtained ~4.8 × 10^6^ utilizable reads for the polyA library, ~4.1 × 10^6^ for the polyC_1_ library, 3.2 × 10^6^ for the polyC_2_ library and only ~3 × 10^5^ reads for the 3’-adapter library (Table 1). For the library based on ligation of a 5’-adenylated RNA adapter to the 3’-end, only ~200 6S–1 RNA-derived pRNA reads were obtained, suggesting that the pRNA sequences were too short for efficient adapter ligation. The distribution of read lengths and RNA classes in the four RNA-Seq libraries is illustrated in Figures S2 and S3, respectively.

**Table 1.** Read numbers of RNA-Seq libraries. Library reads were selected according to the given selection criteria as a measure of library quality. Listed are the total read numbers and the numbers of reads after selection, including the percentage of selected relative to total reads.

Library	Reads total	Selection criteria	Reads after selection	% after selection
polyA	4,866,271	A_5_ plus 3 × A in the next 4 nt	4,848,308	99.6
polyC_1_	4,800,128	C_5_ plus 3 × C in the next 4 nt	4,115,015	85.72
polyC_2_	3,613,711	C_5_ plus 3 × C in the next 4 nt	3,248,713	89.90
3’-adapter	357,487	AGATCGG	302,652	84.6

**Figure 4.**
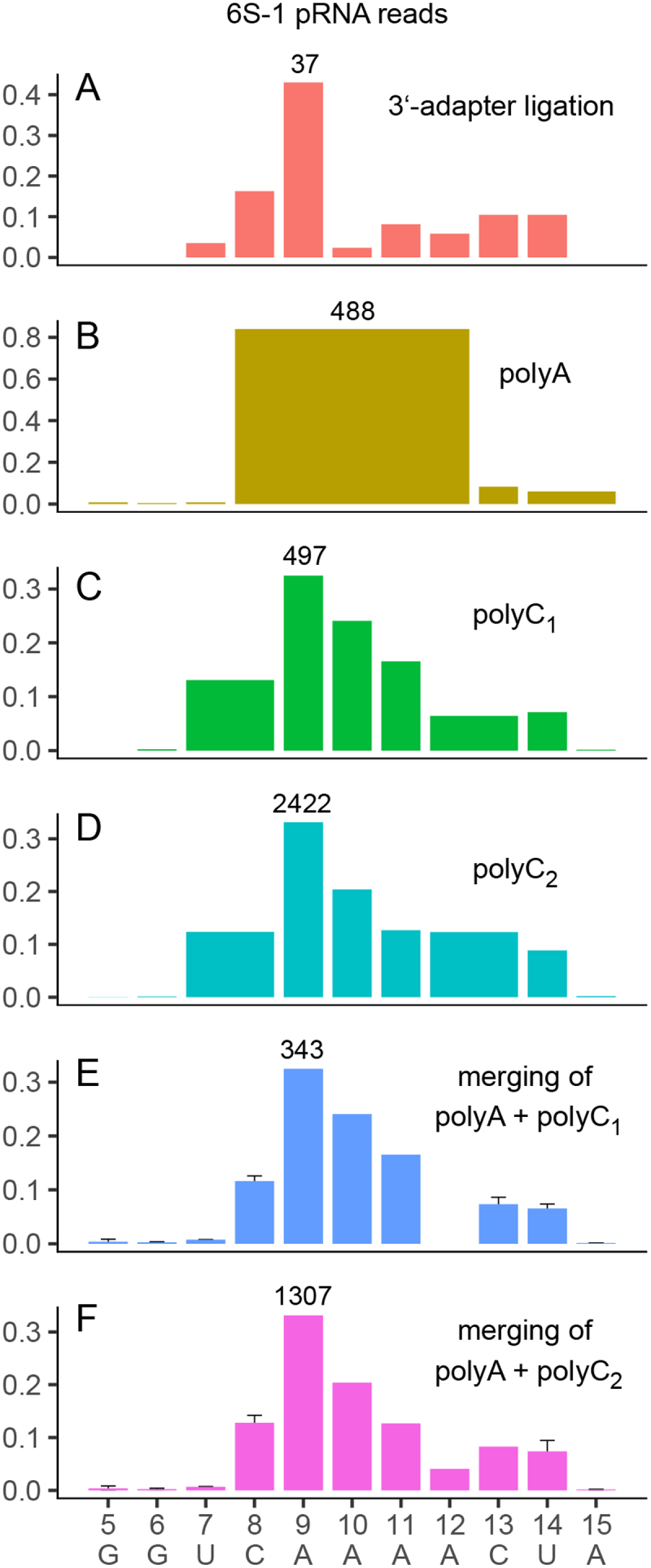
RNA-Seq analysis of pRNAs derived from *B. subtilis* 6S–1 RNA during cell outgrowth from extended stationary phase. (A–D) Length distribution (3’-end variants) of 6S–1 pRNAs in the library constructed via 3’-adapter ligation, and in polyA-, polyC_1_- and polyC_2_-tailing libraries. For details, see text and Tables 1 and 2. (E, F) Numerical fit of read lengths by merging the read profiles for the polyA library with that of the polyC_1_ or polyC_2_ library, as described in Material and methods, illustrated in Figure 5 and detailed in ‘Supplementary Tables pRNA’. The y-axes depict the fraction of reads for the different pRNA length variants in the respective libraries (after read selection, see Table 1). The read number for the most prominent bar is indicated in each profile. All 6S–1 pRNA length variants have identical 5’-ends. Error bars indicate statistical uncertainty of the fitting algorithm.

The 6S–1 pRNA read profiles are shown in Figure 4. Fractionation of small RNAs ≤50 nt as the first step of the library preparation (polyC_2_ library), although resulting in ~20% fewer total reads than in the polyC_1_ library (Table 1), gave rise to almost five times more pRNA reads than in the polyC_1_ library (Table 2). However, the overall length distribution of pRNAs was highly comparable between both preparations (Figure 4(C,D)). Obviously, the fractionation of RNAs ≤50 nt as the first step of library preparation favoured the entry of pRNAs into cDNA libraries.

**Table 2.** Length distribution for 6S–1 pRNA reads in the polyA, polyC_1_ and polyC_2_ libraries. Italic letters in the sequence indicate unresolved nucleotide positions in the context of the respective tailing method. The length distributions are normalized to account for library size differences for each tailing method, such that the sum of all read counts equals one. For example, for 6S–1 pRNA 8- to 12-mers in the polyA library, the normalized count is 488/581 = 0.8399 = 83.99%.

Read count	pRNA length	Sequence	Normalized counts %
PolyA library			
4	5	5’-GTTCG	0.69
2	6	5’-GTTCGG	0.34
4	7	5’-GTTCGGT	0.69
488	8–12	5’-GTTCGGTC*AAAA*	83.99
48	13	5’-GTTCGGTCAAAAC	8.26
35	14–15	5’-GTTCGGTCAAAACT*A*	6.02
Total = 581			
PolyC_1_ library			
3	6	5’-GTTCGG	0.20
200	7–8	5’-GTTCGGT*C*	13.07
497	9	5’-GTTCGGTCA	32.48
368	10	5’-GTTCGGTCAA	24.05
253	11	5’-GTTCGGTCAAA	16.54
98	12–13	5’-GTTCGGTCAAAA*C*	6.41
109	14	5’-GTTCGGTCAAAACT	7.12
2	15	5’-GTTCGGTCAAAACTA	0.13
Total = 1530			
PolyC_2_ library			
1	5	5’-GTTCG	0.01
9	6	5’-GTTCGG	0.12
903	7–8	5’-GTTCGGT*C*	12.34
2422	9	5’-GTTCGGTCA	33.09
1491	10	5’-GTTCGGTCAA	20.37
926	11	5’-GTTCGGTCAAA	12.65
899	12–13	5’-GTTCGGTCAAAA*C*	12.28
647	14	5’-GTTCGGTCAAAACT	8.84
13	15	5’-GTTCGGTCAAAACTA	0.18
2	16	5’-GTTCGGTCAAAACTAG	0.03
1	18	5’-GTTCGGTCAAAACTAGGT	0.01
6	19	5’-GTTCGGTCAAAACTAGGTG	0.08
Total = 7320			

**Figure 5.**
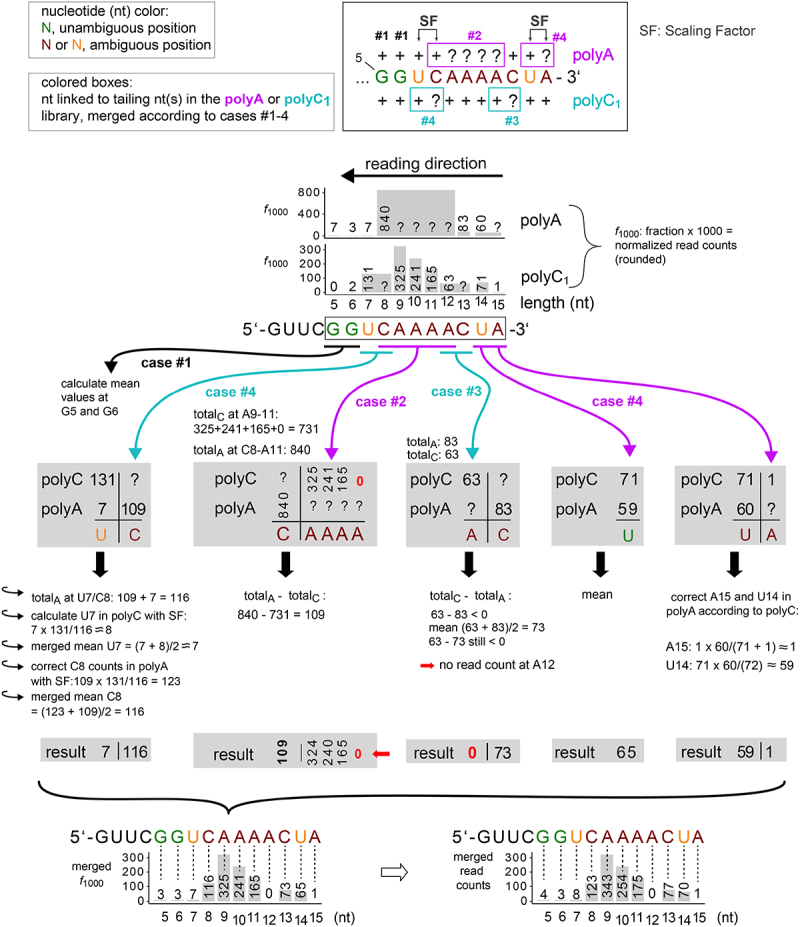
Schematic representation of the numerical fit workflow applied to the distribution of 6S–1 pRNA length variants. The algorithm iterates over the target sequence, from the 3’ end to the 5’ start, and differentiates between four modes, unambiguous case #1 and ambiguous cases #2–4. The four cases are defined and explained under Materials and methods, paragraph ‘Bioinformatics’. The sequence of native pRNA length variants is given from position 5 to 15; green residues indicate unambiguous positions and dark red and orange residues ambiguous positions. In the schematic diagram at the top, question marks indicate unknown position-specific read counts due to A or C trimming of reads; + sign: either unambiguous read counts (cases #1, here G5 and G6) or read counts assigned to the nucleotide 5’ of trimmed nucleotides (e.g. C8 preceding A9–A12 in the polyA library or U7 preceding C8 in the polyC_1_ library) in ambiguous cases #2 to #4; SF, scaling factor. The coloured boxes embrace nucleotides linked to tailing nucleotide(s) in the polyA (magenta) or polyC1 (cyan) library, merged according to cases #1–4. The merged *f*_1000_ values based on combined utilization of the polyA and polyC_1_ libraries are shown at the bottom on the left; the merged read counts at the bottom on the right are obtained by dividing the values for ‘merged *f*_*1000*_’ by 1000 and multiplying with the mean of the sum of read counts from both libraries, (581 + 1530)/2 = 1055.5. As an example, the merged read counts for A9 are (324.84/1000) × 1055.5 = 342.87 = ~343. For more details, see ‘Supplementary Tables pRNA’.

Libraries based on polyA- and polyC-tailing are statistically not directly comparable. Thus, for quantification of the combined data derived from the two tailing approaches, we used a numerical fit that optimizes the Euclidean Distance of the polyA + polyC_1_ and polyA + polyC_2_ data sets (Figure 4(E,F) mergings). The workflow is detailed in Figure 5. The 3’-end distribution for pRNA reads from the library involving 3’-adapter ligation is shown as reference in Figure 4(A). In line with earlier results [9], the majority (84%) of 6S–1 RNA pRNAs represented in the polyA-tailing library were 8- to 12-mers (Table 2, Figure 4(B)). A distinction between 8-, 9-, 10-, 11- and 12-mers was not possible with polyA-tailed fragments due to the run of four A’s at positions 9–12. 8.3% of the pRNA reads represented the 13-mer, 6% 14-/15-mers and only 0.7% the 7-mer. PolyC-tailing allowed us to resolve the stretch of four A residues following the initial eight pRNA nucleotides (5’-GUU CGG UCA AAA-3’). Most pRNAs turned out to be 9-mers (32.5 and 33.1% in the polyC_1_ and polyC_2_ libraries, respectively), followed by 10- and 11-mers (Table 2, Figure 4(C,D)). Conversely, a distinction between 7- and 8-mers as well as 12- and 13-mers was not possible using polyC-tailing alone. Most of the 7-/8-mer reads in the polyC libraries (13.1 and 12.3%) are inferred to represent 8-mers, as polyA-tailing revealed only a few 7-meric pRNAs (0.7%, Table 2). The same pertains to 12-/13-mers that are mainly assignable to 13-mers based on polyA-tailing (8.3% compared with 6.4 and 12.3% for 12-/13-mers in the polyC libraries; Table 2). For the fraction of 14-/15-mers (6%) in the polyA library, polyC-tailing allowed us to assign the vast majority of reads to the 14-mer (Table 2; Figure 4(C,D)). A slight difference between the polyC_1_ and polyC_2_ libraries was an increased proportion of 12-/13-mers and a decrease of 11-mers in the second library (Table 2, Figure 4(C,D)), which may suggest some biological variability of the exact pRNA length pattern if not due to technical variation. The 3’-adapter ligation library (Figure 4(A)) verified the 9-mer as the major pRNA species. A difference between the polyC libraries and the 3’-adapter library is the low fraction of 10-mers in the latter, which should be considered with caution due to the low number of pRNA reads in the 3’-adapter library.

### Other RNA examples in the RNA-Seq libraries

3’-terminal fragments of larger cellular RNAs also entered the libraries, which may originate from intracellular fragmentation, although fragmentation during RNA preparation steps cannot be ruled out. Reads representing 3’-terminal fragments of 6S–1 RNA itself are one example (Figure S4). Here, the polyC and 3’-adapter libraries indicated the major 3’-end of 6S–1 RNA to be GGACUUU-3’, which perfectly matches the previously mapped 3’-end [17,18]. The polyA library is ambiguous at this position, as the 3’-terminal U is followed by an A in 6S–1 RNA precursors (Figure S4, positions 190/191).

3’-terminal fragments of 23S rRNA were represented as well in our libraries (Figure S5(A)). The 3’-end of *B. subtilis* 23S rRNA was previously mapped as UUAACC-3’ [19]. Accordingly, the 3’-adapter library had a single read peak at the second C residue (C2924, Figure S5(A)) and the merging of polyA and polyC libraries also pinpointed UUAACC-3’ as the major 3’-end. The genuine 3’-end of 5S rRNA (CAAGC-3’ [20] was also mapped by merging of the polyA and polyC libraries, consistent with the 3’-adapter read profile (Figure S5(B)).

For some tRNAs, we also identified 3’-end fragments with substantial read numbers in the 3’-adapter library, which allowed a meaningful comparison with polyA and polyC libraries. The isodecoders of tRNA^Asn^ (GUU) (Figure S6) are such an example, where the 3’-adapter library as well as the merging of polyA and polyC libraries indicated the terminal A76 residue as the prevailing 3’-end, as expected. For tRNA^Glu^ (UUC), A_76_ was the prevailing 3’-end in the 3’-adapter library, while C_75_ was the most abundant 3’-terminus according to the merged polyA and polyC libraries (Figure S7(A)). A very similar constellation was observed for tRNA^Gly^ (GCC) (Figure S8(A)). Using our algorithm, we also simulated the polyA/(C)-libraries for tRNA^Glu^ (UUC) and tRNA^Gly^ (GCC) under the virtual assumption that the 3’-adapter libraries conveyed the correct picture. Comparison of the simulated (Figure S7(B), S8(B)) and experimental (Figure S7(A), S8(A)) read profiles illustrates the strongest differences in the polyC profiles, implying that 3’-truncated tRNA fragments are more represented in polyC libraries than in 3’-adapter libraries. These observations suggest that 3’-end fragments of tRNAs, which may include part of the tRNA acceptor stem, are utilized somewhat differently as substrates in 3’-adapter ligation than in C-tailing reactions by polyA polymerase, whatever the reasons for this difference might be.

## Discussion

We have presented a methodological framework for RNA-Seq studies focussing on very short RNAs, involving protocols for polyA-, C- and U-tailing that can be used as starting points after RNA size fractionation and in preparation for reverse transcription reactions. Our approach was motivated by the inefficient inclusion of very short RNAs in RNA-Seq libraries based on 3’-adapter ligation. The experimental and data analysis framework presented herein allows one to fully resolve the distribution of 3’-end variants of ultrashort RNAs down to the single nucleotide level in RNA-Seq applications, based on sufficiently high read numbers. This was achieved by 3’-tailing with two different nucleotides, here by combining polyA- with polyC-tailing, and applying a numerical fit approach to assess 3’-end distributions at sequence positions where a nucleotide of different identity than the tailing nucleotide is followed by one or more residues with the same base identity as the respective tailing nucleotide. We showed that polyU-tailing is an additional option and even oligoG-tailing with polyA polymerase from yeast was previously applied with success, in this case to investigate the length of polyA tails of viral mRNAs using oligo(dC/T) reverse transcription primers against the polyA/oligoG-tailed 3’-end region [21].

Our analysis revealed 9-mers as the largest fraction of 6S–1 pRNA reads and 13/14-mers as the longest abundant variants (Figure 4). This finding suggests striking parallels to the *E. coli* system, where two pRNA length variants were found to play key roles in a mechanistic *in vitro* study: pRNA 9-mers that triggered the release of σ^70^ from RNAP and 13-mers resulting in 6S RNA dissociation from core RNAP [14]. Our findings suggest that the 6S–1 RNA release mechanism from *B. subtilis* RNAP equals that of *E. coli* 6S RNA from its cognate RNAP, in that a 9-mer triggers σ^A/70^ release and further elongation to 13- or 14-mers triggers dissociation of the RNA from core RNAP. As demonstrated for the *E. coli* system and assumed to also apply to *B. subtilis*, synthesis of a pRNA 9-mer results in ejection of σ^A/70^ from RNAP in a ‘scrunching’-type release mechanisms that reports a switch of the enzyme from abortive transcription to the elongation mode. However, elongation complexes cannot proceed as processively as at DNA promoters and terminate at a pRNA length of 13 to 14 nt owing to the refolding of 6S RNA that destabilizes the interactions with core RNAP [10,14], making the synthesis of longer run-off pRNA transcripts a rare event [9,22]. In view of these suggested mechanistic parallels between *E. coli* and *B. subtilis* RNAP, it is interesting to note that a specific detail of pRNA-induced refolding of *E. coli* 6S RNA and *B. subtilis* 6S–1 RNA is different: the former forms a hairpin structure in its central region, while the latter forms a so-called central bulge collapse helix [10,14], in addition to the basic structural rearrangement caused by pRNA invasion into the 6S RNA structure. The abundance of pRNA 13-/14-mers was lower than that of 9-mers in all libraries (Figure 4). Considering that the dissociation rate from 6S–1 RNA will be lower for 13-/14-mers than 9-mers, the equilibrium of dissociated pRNAs and those bound to 6S–1 RNA will be shifted to the bound state for 13-/14-mers. As a consequence, the 6S–1 RNA-bound pRNAs may have been less accessible to 3’-end ligation or tailing and therefore remained underestimated. Thus, the fractions of 13-/14-mers should be considered as a lower limit. We are also aware that the pRNA length distribution *in vivo* may not necessarily be simply a blueprint of the pRNAs synthesized by RNAP on 6S RNA as template; we cannot exclude that pRNA length variants are degraded by cellular RNase with different kinetics, possibly altering the steady-state length distribution of pRNAs in live cells. A *B. subtilis* strain with knockouts of four 3’-exonucleases showed increased levels of 6S–1 pRNA 13-/14-mers [18], illustrating that 3’-exonucleolytic degradation of pRNAs contributes to their steady-state levels in bacterial cells.

As yet there is no high-resolution RNA-Seq data available for the *E. coli* system to assess if 9- and 13-mers are also major pRNA species in *E. coli* cells during outgrowth. Based on early *in vitro* studies, pRNAs in the *E. coli* system were reported to be 14 to 24 nt in length [8,13]. As the first 16 nt of pRNAs derived from *E. coli* 6S RNA are 5’-AUC GGC UCA GGG GAC U-3’, our approach will be suitable to resolve 8-/9-mers and 13-/14-mers in future attempts to analyse growth phase-dependent pRNA length patterns in *E. coli* cells. Additionally, polyU- or polyG-tailing may be considered to substantiate 3’-end distribution in C/A-rich regions of pRNAs.

*B*. *subtilis* 6S–1 pRNA 9-mers include the first A residue of the A_4_ homonucleotide stretch (6S–1 pRNA 14-mer: 5’-GUU CGG UCA AAA CU-3’); 9-mers transcribed *in vitro* from *E. coli* 6S RNA as template are terminated immediately before addition of a G_4_ homonucleotide stretch (see sequence above). *Aquifex aeolicus* 6S RNA encodes pRNAs with a C_4_ stretch at position 6 to 9 (5’-GUA GGC CCC AUU GAC AA-3’ [23]. This suggests the possibility that such homonucleotide stretches decelerate the rate of NTP incorporation and thereby finetune the process of 6S RNA release from RNAP. Thus, for carving out mechanistic commonalities and differences in the regulation of 6S RNA function in different bacteria, it will be instructive to analyse their pRNA length patterns as a function of the cellular nutrient/energy state applying our methodology presented here.

Other applications of our approach may include short transcripts derived from abortive transcription by viral or other polymerases; for example, the Ebola viral RNAP synthesizes such abortive transcripts of varying length at its leader promoter [24]. 3’-end tailing may counteract underrepresentation of shorter abortive transcript species in RNA-Seq libraries employing 3’-adapter ligation. Also, analysis of *in vivo* length profiles of short abortive transcripts synthesized at bacterial DNA promoters in the context of mechanistic investigations may be of interest, as could be the detection of shorter oligoribonucleotide end products derived from endo and exoribonucleolytic RNA processing reactions. For RNA-Seq analysis of abortive promoter transcripts, we recommend to adapt the approach when investigating a specific promoter that is sufficiently active and known from *in vitro* studies to be prone to abortive transcription. We are aware of the possibility that cellular levels of abortive promoter transcripts may not reach those of 6S–1 RNA-derived pRNAs. In cases of non-detectability of such short RNAs by RNA-Seq, follow-up RNA-Seq analyses with different amounts of near-isosequential spike-in RNA oligonucleotides mimicking the abortive promoter transcripts could be helpful in assessing and improving the detection limit for the RNAs of interest, e.g. by further enrichment of short RNAs. Furthermore, short eukaryotic RNAs including miRNAs may be analysed in addition to protocols utilizing 3’-adapters to avoid that the levels of certain miRNAs are underestimated owing to reduced efficiency of 3’-adapter ligation. In conclusion, we have provided a framework for RNA-Seq of ultrashort RNAs that addresses – and sensitizes the reader to – several idiosyncrasies of this class of RNAs. Only minor adaptations are required to apply the methodology to other short RNAs of bacterial, archaeal, eukaryal or viral origin.

Another application that we immediately noticed are tRNA fragments commonly found in cancer cells but also in healthy tissues. For example, three series of tRNA fragments (tRFs) were identified in a prostate cancer cell line, of which series tRF-3 varied in length from 13 to 22 nucleotides [25]. Particularly the shorter ones are likely to be incorporated more efficiently into RNA-Seq libraries constructed by 3’-tailing. For 3’-proximal tRFs, it may be advantageous to explore polyU (or polyG- tailing), taking into account that our 3’-end profiles for tRNA 3’-end fragments varied between RNA-Seq libraries based on 3’-adapter ligation versus 3’-polyC-tailing (see last paragraph of the Results section and Figure S7, S8).

Finally, for determining the 3’-end profiles of short cellular RNAs by RNA-Seq, ultimate controls would be spike-ins of mixtures of short synthetic RNA length variants mimicking those of interest. Such spike-ins would be added to RNA extracts from derivative cells with knockouts of the gene under investigation, or they may be designed with e.g. 2 internal nt exchanges relative to the native RNA fragments of interest to enable their specific identification. For studies on tRFs and before performing RNA-Seq with cellular RNA extracts, mixtures of synthetic RNAs mimicking the expected tRF length spectrum could be directly subjected in parallel to 3’-end ligation of the 5’-adenylated RNA adapter and 3’-poly(A/U/C/G)-tailing, followed by RNA-Seq analysis to assess the representation of individual length variants in the corresponding read libraries.

## Materials and methods

### *In vitro* poly(A/C/U)-tailing

For polyA-tailing, we used a recombinant polyA polymerase from *E. coli* (New England Biolabs [NEB]). Assays were performed with 1 µM of a synthetic *B. subtilis* 6S–1 pRNA 8-mer (5’-GUU CGG UC-3’) or 14-mer (5’-GUU CGG UCA AAA CU-3’) (both synthesized by NOXXON Pharma AG, Berlin) containing trace amounts of the same respective 5’-^32^P-labelled RNA oligonucleotide (10,000 Cherenkov cpm), 1 mM ATP, 4 mM DTT and 5 units polyA polymerase in 1× reaction buffer (50 mM Tris-HCl, 250 mM NaCl, 10 mM MgCl_2_, ~pH 8.0). The final reaction volume was 10 µl. If MnCl_2_ was added for higher tailing efficiency, we used 2.5 mM as used previously [26]. Samples were incubated for 2 h at 37°C and stopped by adding denaturing gel loading buffer (2.6 M urea, 66% (v/v) formamide, 0.02% (w/v) bromophenol blue, 0.02% (w/v) xylene cyanol blue, 2× TBE). Samples were heated to 98°C for 3 min, followed by immediate cooling on ice before RNA separation using 20% denaturing (8 M urea) polyacrylamide gel electrophoresis (PAGE) with 1× TBE as running buffer. Radioactive RNAs were visualized using the BIO-Imaging Analyzer FLA 3000-2 R (FUJIFILM) and phosphor images were processed utilizing the AIDA (raytest) image analysis software.

For polyC-tailing, we used *E. coli* polyA polymerase (NEB), the PolyA Polymerase Tailing Kit (Lucigen) and the A-Plus PolyA Polymerase Tailing Kit (Cellscript) in the basic 1× reaction buffer containing 50 mM Tris-HCl, 250 mM NaCl, 10 mM MgCl_2_ (~pH 8.0) supplemented with 4 mM DTT. To optimize tailing efficiency, we varied the CTP concentration (1 mM or 3 mM) and added, if indicated, MnCl_2_ to a final concentration of 2.5, 5 or 10 mM. Furthermore, we incubated the reaction for 2 or 4 h at 37°C, or 2 + 2 h with another 1 µl-aliquot of polyA polymerase added to the 10 µl reaction volume after the first 2 h of incubation. Processing of samples after incubation was performed as described above for polyA-tailing.

To elongate short RNAs with UTP, a PUP (NEB; the *Schizosaccharomyces pombe* Cid1 gene product recombinantly expressed in *E. coli*) and the corresponding reaction buffer (1× NEBuffer 2: 50 mM NaCl, 10 mM Tris-HCl, 10 mM MgCl_2_, 1 mM DTT, pH 7.9) were used. PolyU-tailing was performed with 1 µM of the synthetic pRNA 8- or 14-mer (see above) containing trace amounts of the same respective 5’-^32^P-labelled pRNA (10,000 Cherenkov cpm), 1 mM UTP, and indicated units of PUP in the reaction buffer specified above. No extra DTT was added, as the reagent was already introduced with the reaction buffer. The samples were incubated for 2 or 4 h at 37°C, or for 2 + 2 h which involved addition of another aliquot of PUP after the first 2 h of incubation.

### Bacterial growth and Northern blot analysis

*B*. *subtilis* PY79 wild-type cells were grown in LB medium at 37°C under gentle shaking (200 rpm, Aquatron water bath shaker, Infors AG, Germany). Complete growth curves were recorded (by optical density measurements at 600 nm = OD_600_) using cultures containing 500 ml LB medium and inoculated with an overnight culture to a starting OD_600_ of 0.05. Cell aliquots were harvested at several time points of the growth curve. To induce outgrowth, cell aliquots from early or extended stationary phase were diluted 1:5 in fresh, prewarmed LB medium. Three minutes after initiation of outgrowth, samples of 50 ml were withdrawn for RNA isolation. For cells harvested in exponential phase, only ~7 ml were withdrawn for RNA isolation, yielding sufficient quantities of RNA for the Northern blot analysis. Total cellular RNA for Northern blot analysis of 6S–1 RNA-derived pRNAs was isolated from frozen cell pellets using a phenol extraction method consisting of a hot phenol and a cold phenol step [27,28].

For pRNA detection via Northern blotting, 6 µg of total RNA was loaded on a 10% native polyacrylamide (PAA) gel. As size marker, the chemically synthesized pRNA 14-mer (see above) was 5’-phosphorylated using T4 polynucleotide kinase (Thermo Scientific Fermentas) before gel loading (0.25, 1 and 5 ng). For details see [27] and [15].

### RNA sequencing (RNA-Seq)

RNA integrity was examined by capillary electrophoresis before preparation of cDNA. Starting from total RNA samples, small RNA species <200 nt were enriched with the mirPremier microRNA Isolation Kit (Sigma Aldrich); for the second polyC library, termed polyC_2_ library, total cellular RNA was alternatively fractionated by preparative denaturing 15% polyacrylamide gel electrophoresis. RNAs ≤50 nt were localized by coelectrophoresis of a 50 nt long oligonucleotide and eluted from the gel. After these fractionation steps, RNAs were either polyA-tailed, polyC-tailed or a specific 5’-adenylated RNA adapter was ligated to the 3’-end. For polyA-tailing, 200 ng of the small RNA fraction were incubated for 5 min at 37°C with 2.5 U *E. coli* polyA polymerase (NEB) in a reaction volume of 25 µl containing 1 mM ATP and 1× reaction buffer (250 mM NaCl, 50 mM Tris-HCl, 10 mM MgCl_2_, pH 7.9). For polyC-tailing, 200 ng of the small RNA fraction were incubated for 2 h at 37°C with 5 U *E. coli* polyA polymerase (NEB) in 1× reaction buffer (see above) supplemented with 1 mM CTP (instead of ATP), 4 mM DTT and 2.5 mM MnCl_2_ (reaction volume 10 µl); after the first 2 h of incubation, another 5 U (1 µl) of polyA polymerase were added followed by incubation for another 2 h (performed as in Figure 1(B), lane 3, and Figure 1(C), lane 6). After polyA- or polyC- tailing, the RNA was treated with tobacco acid pyrophosphatase (TAP, Epicentre) to convert 5’-triphosphates to 5’-monophosphates, followed by 5’-adapter ligation (TruSeq Universal Adapter, with a phosphorothioate linkage between the two 3’-terminal residues). First-strand cDNA synthesis was either conducted with the TruSeq antisense oligo (dT)-adapter primer [polyA-tailing] or the TruSeq antisense oligo(dG)-adapter primer [polyC-tailing] using Moloney Murine Leukemia Virus (M-MLV) reverse transcriptase (see Table 3 for adapter and primer sequences). In the case of 3’-adapter ligation, a 5’-adenylated RNA adapter (TruSeq Indexed Adapter) was first ligated to the 3’-end of the RNAs, followed by TAP treatment to remove 5’-triphosphates. Then the TruSeq Universal Adapter was ligated to the 5’-ends. First-strand cDNA synthesis using M-MLV reverse transcriptase was performed with the correspondingly bar-coded TruSeq Indexed Antisense Primer. The resulting cDNAs were PCR-amplified to about 20–30 ng/µl using a high-fidelity DNA polymerase (such as Q5 High-Fidelity DNA polymerase from NEB; for a recent comparison of high-fidelity DNA polymerases, see [29], using PCR primers 1.0 and 2.0 (Table 3) and conducting 15 PCR cycles for the polyA library, 22 for the polyC_1_ library, 21 for the 3’-adapter library and 19 for the polyC_2_ library. The combined lengths of the non-native flanking sequences (excluding the 25 dT or 15 dG nucleotides encoded in the adapter primers) was 121 nt in all three approaches. The PCR reaction mixtures containing the cDNA libraries were purified using the Agencourt AMPure XP kit (Beckmann Coulter Genomics) and analysed on a Shimadzu MultiNA microchip electrophoresis system. To further enrich for cDNAs corresponding to RNAs ≤50 nt in the case of polyA, polyC_1_ and 3’-adapter libraries, the cDNA samples were size-fractionated by preparative 6% PAGE gels, eluting cDNAs smaller than ~200 bp (polyA library), ~190 bp (polyC_1_ library) or ~ 160 bp (3’-adapter library). Aliquots of the size-fractionated cDNAs were analysed by capillary electrophoresis. Library construction and single-end sequencing (75 nt read length) using an Illumina sequencer was carried out by Vertis Biotechnologie AG (Freising, Germany).

**Table 3.** Adapters and primers used for library construction and Illumina sequencing.

5’-adapter (TruSeq Universal Adapter) 5’-AATGATACGGCGACCACCGAGATCTACACTCTTTCCCTACACGACGCTCTTCCGAT C*T-3’ (*: phosphorothioate linkage) TruSeq antisense oligo(dT)-adapter primer for cDNA synthesis in polyA-tailing *Barcode* 5’-CAAGCAGAAGACGGCATACGAGAT-NNNNNN-GTGACTGGAGTTCAGACGTGTGCTC TTCCGATC(dT)_25_-3’ TruSeq antisense oligo(dG)-adapter primer for cDNA synthesis in polyC-tailing *Barcode* 5’-CAAGCAGAAGACGGCATACGAGAT-NNNNNN-GTGACTGGAGTTCAGACGTGTGCTC TTCCGATC(dG)15–3’ 5’-adenylated RNA adapter (TruSeq Indexed Adapter) used for 3’-adapter ligation *Barcode* 5’-rApp-GATCGGAAGAGCACACGTCTGAACTCCAGTCAC‐NNNNNN‐ATCTCGTATGCCG TCTTCTGCTTG-3’ TruSeq Indexed Antisense Primer for cDNA synthesis, complementary to ligated 3’-adapter *Barcode* 5’-CAAGCAGAAGACGGCATACGAGAT-NNNNNN-GTGACTGGAGTTCAGACGTGTGCTC TTCCGATC-3’ PCR Primer 1.0 5’-AATGATACGGCGACCACCGAGATCTACACTCTTTCCCTACACGA PCR Primer 2.0 5’- CAAGCAGAAGACGGCATACGAGAT

### Bioinformatics

The RNA-Seq read libraries were trimmed according to the specific tailing methods at the first stretch of five consecutive adenosines or cytidines followed by at least four nucleotides, of which at least three are adenosines or cytidines, respectively. For the adapter-ligated library, trim_galore (v0.6.7) with cutadapt (v4.1) was used with a minimal length cut-off of four (–length 4) and disabled error rates (−e 0). The reads were then mapped to the selected target sequences, such as the 6S–1 pRNA, 6S–1 RNA, tRNAs, etc., ensuring an overlap of the first 30 of the last (3’-proximal) 40 nucleotides of the target sequence without any mismatches. For example, in the case of 6S–1 RNA (Figure S4), the 3’-proximal 11 nt of the 40-nt window were quantitatively compared for 3’-end coverage and the 5’-proximal 30 nt were used for the mismatch-free alignment of reads. For targets that are shorter than 40 nucleotides (6S–1 pRNAs), the first (5’-proximal) five nucleotides were used instead as the selection criterion. For each target, read lengths from the trimmed libraries were aggregated into length distributions.

A schematic representation of the workflow is shown in Figure 5 for the 6S–1 pRNA libraries. The algorithm iterates through the target sequence, from the 3’ end to the 5’ start, and differentiates between four cases. Unambiguous cases #1 (marked by dark green positions in the pRNA sequence in Figure 5) applies to nucleotide positions that have another base identity (i.e. G or U/T) than the tailing nucleotide (A or C) and that are either contiguous to a non-tailing nucleotide (G or U) on their 3’ side, or that are contiguous to a tailing nucleotide (A or C) that was determined as 0 in the preceding step (see example of U65 in Supplementary Tables tRNA-Glu). The output is calculated by the normalized read counts at this position averaged over the polyA and polyC libraries. U residues followed by an A or C residue (with an *f*_1000_ > 0) on their 3’ side are shown in orange. Standard deviations (error bars in Figure 4 and related figures) indicate positional deviations between the merged libraries.

Ambiguous cases arise where one or more 3’-terminal RNA nucleotides have the same identity as the tailing nucleotide, and are consequently trimmed during processing of the cDNA libraries. Ambiguous positions (dark red letters in Figure 5) are handled according to cases #2, #3 or #4. The ambiguous case #2 applies to constellations where an ambiguous case in one library runs on its 5’ side into an ambiguous position in the other library, which is the hallmark of case #2. This can be seen in the diagram at the top of Figure 5 for the four consecutive A residues (A9-A12) that are trimmed during data processing such that all read counts are assigned to C8 (C8 to A12 encased by the purple box) in the polyA library; yet, position C8 itself is ambiguous in the polyC library (marked by the question mark in the cyan box). For A9-A12, the normalized read counts (also termed ‘fraction of reads × 1000’ or ‘*f*_1000_’, see Supplemental Tables pRNA) from the polyC-tailed library are used to assign read counts for all positions in this window. The normalized read count assigned to the nucleotide immediately 5’ of the four A’s that differs from adenosine (position C8 in the polyA-tailed library) is defined as total_A_. The accumulated values for the polyC library in this window (with value 0 for position 12 as determined in the preceding ambiguous case; see below) are defined as total_C_. The value of the polyA library at position C8 is corrected by subtracting the total_C_ value from the total_A_ value (total_A_ ─ total_C_). For the homopolymer stretch of the 4 A’s in Figure 5, the total_C_ value is 731 = 325 + 241 + 165 + 0 (sum of normalized counts for positions 9–12) and total_A_ is 840, such that the corrected count for C8 in the polyA library is 840–731 = 109. This value is further balanced by a scaling factor (SF) in a case #4 operation (see below) that averages total_A_ and total_C_ for the interconnected positions C8 and U7.

Case #3 is conceptually like case #2, but with a basic incongruency between the libraries, exemplified by A12/C13 (see top diagram in Figure 5). At position C13 of the pRNA sequence, 83 counts are unambiguously assigned to C13 in the polyA library, while 63 counts in the polyC library have to be distributed among A12 and C13. A correction as above (total_C_ − total_A_) results in a negative value (63–83 = − 20) for A12 in the polyC library. Thus, A12 is corrected to 0 based on the polyC library and the average between (63 + 83)/2 = 73 is assigned to C13 instead. In summary, the hallmark of case #3 is the generation of negative numbers during the merge of fractional read counts. In such cases, the algorithm issues a user warning to indicate an incongruency between the libraries.

Ambiguous case #4 allows for a local renormalization as all the information is contained at these positions. The condition for case #4 is met when the 5’-nucleotide of an ambiguity window is a non-tailing nucleotide (U or G). In the diagram at the top of Figure 5, this applies to A15/U14 and C8/U7. Here a question mark in one library faces a plus sign in the other library, and plus signs in both libraries are present 5’ of the position with question mark. Exemplified for A15/U14, the idea is that the counts in the polyC library should represent the true distribution of reads as no C nucleotide is part of this subsequence (here A15 ~ 1 normalized count, U14 ~ 71 normalized counts). To integrate the partial information of the polyA library that only represents the total count of this dinucleotide sequence (60 normalized counts), the counts for A15 in the polyA library are estimated with a SF that takes account of the difference in total_A_ and total_C_ for positions U14/A15 in the two libraries: the normalized count of ~ 1 for A15 in the polyC library is multiplied with the SF of 60/(71 + 1) = ~1, thus assigning 1 normalized count to A15 in the polyA library; correspondingly, the normalized counts for U14 in the polyA library are calculated as 71 × 60/72 = ~59 (Figure 5). Then the scaled polyA counts and the original polyC counts are averaged at these positions to form the resulting distribution of counts (mean for A15 = ~1; mean for U14 is (71 + 59)/2 = 65).

As mentioned before, the algorithm iterates through the entire sequence from 3’ to 5’, applying these rules to merge the libraries. The SFs are used to ensure that the sum of read fractions remains 1 in the merged libraries. The detailed calculation for the pRNA sequence can be found in the Supplement (Supplementary Tables pRNA). The difference between A15/U14 and C8/U7 is that A15/U14 represents an ab initio case #4, while C8/U7 becomes a case #4 after the *f*_1000_ value for C8 in the polyA library has been calculated with the help of the polyC library in the preceding step. If C8 turned out to be case #3 (*f*_1000_ at C8 = 0), then U7 would be treated as case #1 (see example of U65 in Supplementary Tables tRNA-Glu).

**Scheme 1.**
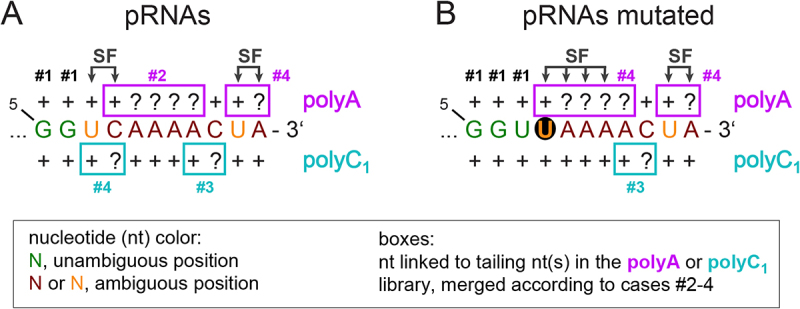
Merging of read counts for 6S–1 pRNAs from polyA and polyC_1_ libraries. (A) Schematic sequence diagram for native pRNA length variants from position 5 to 15. Question marks: unknown position-specific read counts due to A or C trimming of reads; + sign: either unambiguous read counts (cases #1, here G5 and G6) or read counts assigned to the nucleotide 5’ of trimmed nucleotides (e.g. C8 preceding A9-A12 in the polyA library or U7 preceding C8 in the polyC_1_ library) in ambiguous cases #2 to #4; SF, scaling factor. (B) Mutated pRNA sequence where the sequence was changed from C8 to U8 and the 200 read counts for U7/C8 in the polyC_1_ library (Table 2) arbitrarily distributed between U7 and U8 (10 assigned to U7, 190 to U8).

The difference between case #2 and #4 is further illustrated in Scheme 1 where we artificially changed C8 of the pRNA sequence to U8 (Scheme 1(B)). With this mutated sequence, the algorithm now treats the 4 A’s as case #4, because there is no ambiguity anymore on the 5’ side of A9-A12. The sum of read fractions from U8 to A12 are then averaged for the polyA and polyC_1_ libraries by use of a SF. For details of the calculations (using a pocket calculator) and output protocols of the algorithm, see the Supplement (Supplementary Tables pRNA).

We also simulated polyA/(C) libraries based on the experimental 3’-adapter library to validate the method, exemplified for tRNA^Glu^ (UUC) ending with CCA_76_ (Figure S7(B)). The corresponding 3’-end distribution of the experimental 3’-adapter library at C_74_, C_75_ and A_76_ is 1, 21 and 133 – representing 1 read ending with C_74_, 21 reads ending with C_75_, and 133 reads ending with A_76_. Now consider the simulated polyA library. Since reads ending with C_74_ are unambiguous in the experimental polyA library (Supplementary Tables tRNA-Glu simulated), we can assign the single read of the 3’-adapter library to C_74_, multiplied by the total read count (12038) in the experimental polyA library and divided by the total read count (212) of the 3’-adapter library: 1 × 12038/212 = 56.783 = ~57. This is the SF for the polyA library. As reads ending at C_75_ and A_76_ are ambiguous in the polyA library, we sum up the read counts for these positions in the 3’-adapter library (21 + 133 = 154) and multiply this number with the SF: 154 × 56.783 = 8745, corresponding to a fraction of 0.726 ( = 8745/12038) covering positions 75 and 76 (Figure S7(B), Supplementary Tables tRNA-Glu simulated). The same principle is adapted to positions 66 to 73 (the 3’-adapter library had no reads at A_66_ or further upstream). For the polyC_1_ library (total reads: 1396) reads ending with A_76_ are unambiguous. Thus, the 133 reads for this position in the 3’-adapter library can be multiplied with the SF for the polyC_1_ library, namely 1396/212 = 6.585 × 133 = 876. Positions 73–75 are ambiguous in the polyC_1_ library, thus we sum up the read counts for these positions in the 3’-adapter library, 0 (A_73_) + 1 (C_74_) + 21 (C_75_) = 22, multiplied by the SF 6.585, yielding a simulated read count for A_73_ to C_75_ of 145, corresponding to a fraction of 0.104 ( = 145/1396), covering positions 73 and 75 (Figure S7(B)). The same principle is adapted to positions 66 to 72. The merging of the simulated polyA and polyC_1_ libraries is performed as detailed in ‘ Supplementary Tables tRNA-Glu simulated’.

## Supplementary Material

Damm_Klemm_R1_260225_SM cleaned.docx

## Data Availability

All scripts, plots and data sets generated for this study can be found in the uni marburg GitLab repository (https://gitlab.uni-marburg.de/synmikro/ag-lechner/3-tailing-method-for-ultrashort-rnas). The raw sequencing files from this study are available at the NCBI Sequence Read Archive (SRA) under BioProject PRJNA1158698, BioSample accessions SRR30607193, SRR30607194, SRR30607195, SRR30607196.
