# Supplementary material for: 6S-1 pRNA 9-mers are a prominent length species during outgrowth of Bacillus subtilis cells from extended stationary phase
Source: RNA Biol. 2025 Mar 25;22(1):1–14. doi: 10.1080/15476286.2025.2484519 (PMC12005410; doi:10.1080/15476286.2025.2484519)
Supplement: Damm_Klemm_R1_260225_SM cleaned.docx [file KRNB_A_2484519_SM3574.docx]

**SUPPLEMENTARY MATERIAL**

**6S-1 pRNA 9-mers are a prominent length species during outgrowth of *Bacillus subtilis* cells from extended stationary phase**

Katrin Damm^1,§^, Paul Klemm^2,§^, Marcus Lechner^2^, Dominik Helmecke^1^ and Roland K. Hartmann^1,^*

^1^ Institut für Pharmazeutische Chemie; Philipps-Universität Marburg; Marburg, Germany

^2^Center for Synthetic Microbiology (SYNMIKRO), Philipps-Universität Marburg, Marburg, Germany

E-mail addresses:

katrin-damm@gmx.de

klemmp@staff.uni-marburg.de

lechner@staff.uni-marburg.de

helmecke@staff.uni-marburg.de

*Corresponding author: E-mail: [roland.hartmann@staff.uni-marburg.de](mailto:roland.hartmann@staff.uni-marburg.de)
^§^These two authors contributed equally to this work

**
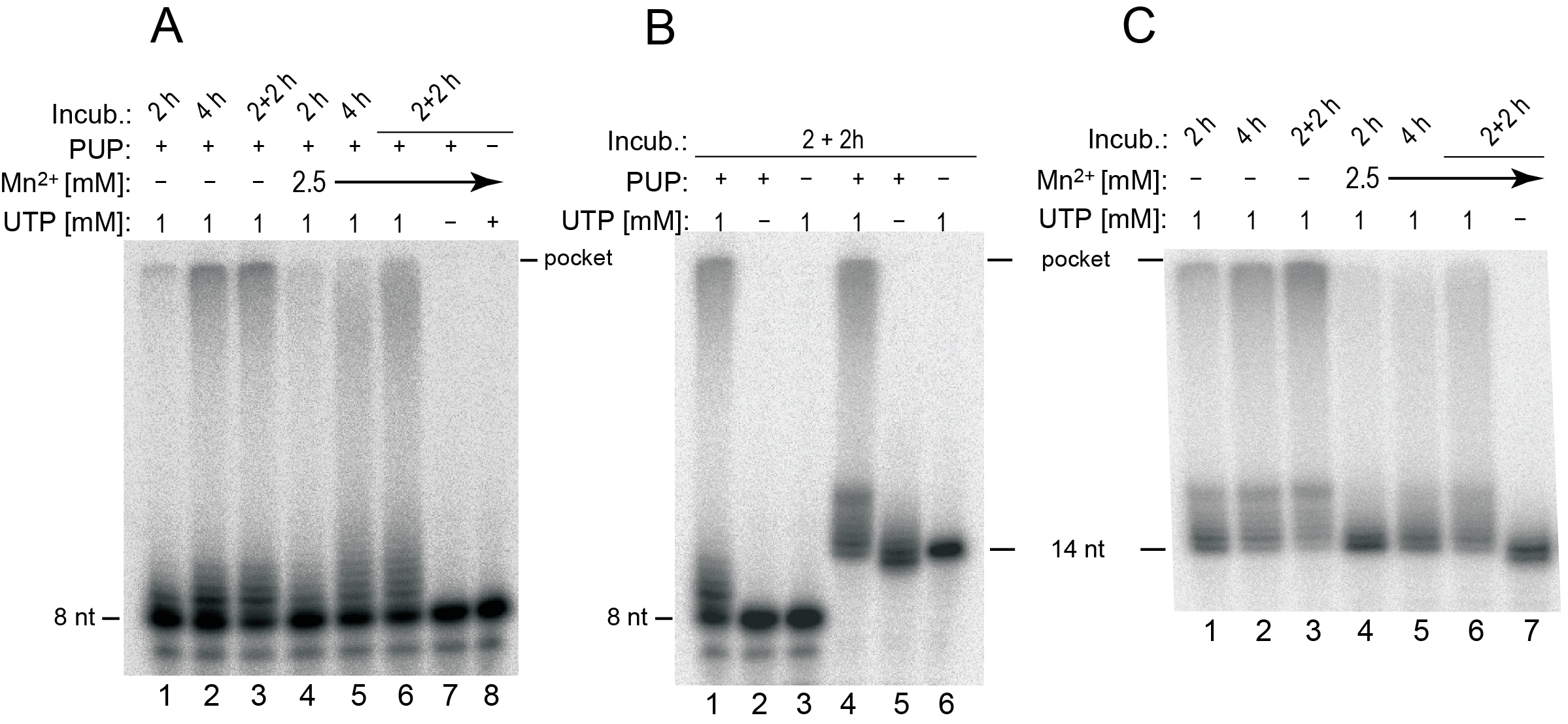
Supplementary Figures**

**Fig. S1:** Effect of Mn2+ on polyU tailing of the RNA 8- and 14-mer by polyU polymerase. 1 µM RNA 8-mer (**A**) or 14-mer (**C**) containing trace amounts of the sequence-identical 5’-32P-labeled RNA (10,000 Cherenkov cpm) were incubated in 1× NEBuffer 2 (see Materials and methods of the main manuscript) with polyU polymerase (PUP) and 1 mM UTP, either in the presence or absence of 2.5 mM MnCl_2_. Incubation was for 2 or 4 h at 37°C using 2 U of enzyme, or after 2 h of incubation in the presence of 2 U enzyme another 2 U were added followed by another 2 h of incubation at 37°C (2+2 h). In panel C, enzyme was added to all samples. (**B**) Side by side analysis of polyU tailing of 8- and 14-mer in the absence of Mn2+; 2+2 h incubation: as described for panel A. The shown gels were of the same type (20% urea PAGE, identical gel dimensions and electrophoresis conditions) as in Fig. 1D, E of the main text.


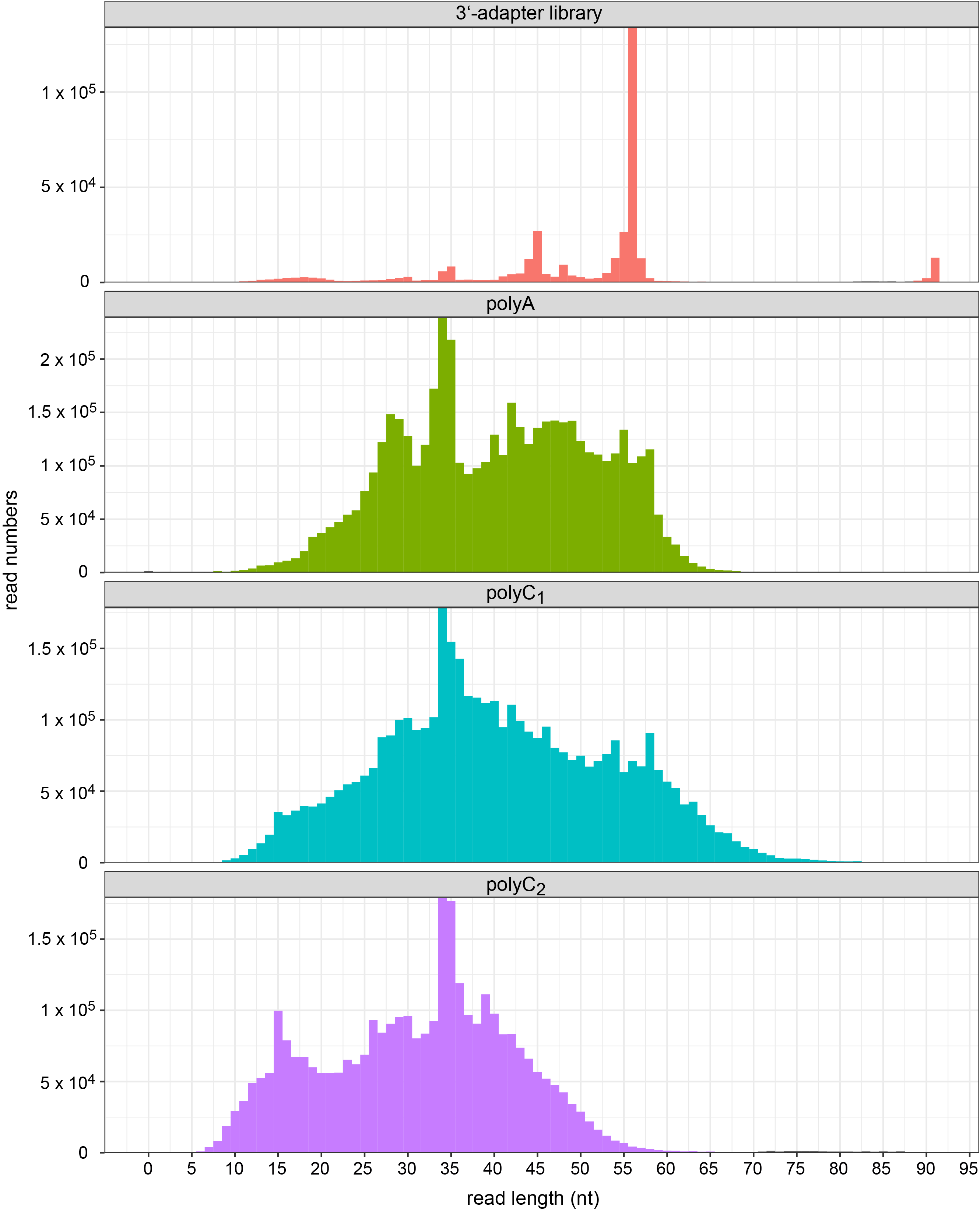


**Fig. S2:** Distribution of read lengths in the four RNA-seq libraries. For example, reads with a length of 56 nt were the most frequent length variant in the 3’-adapter library.

**
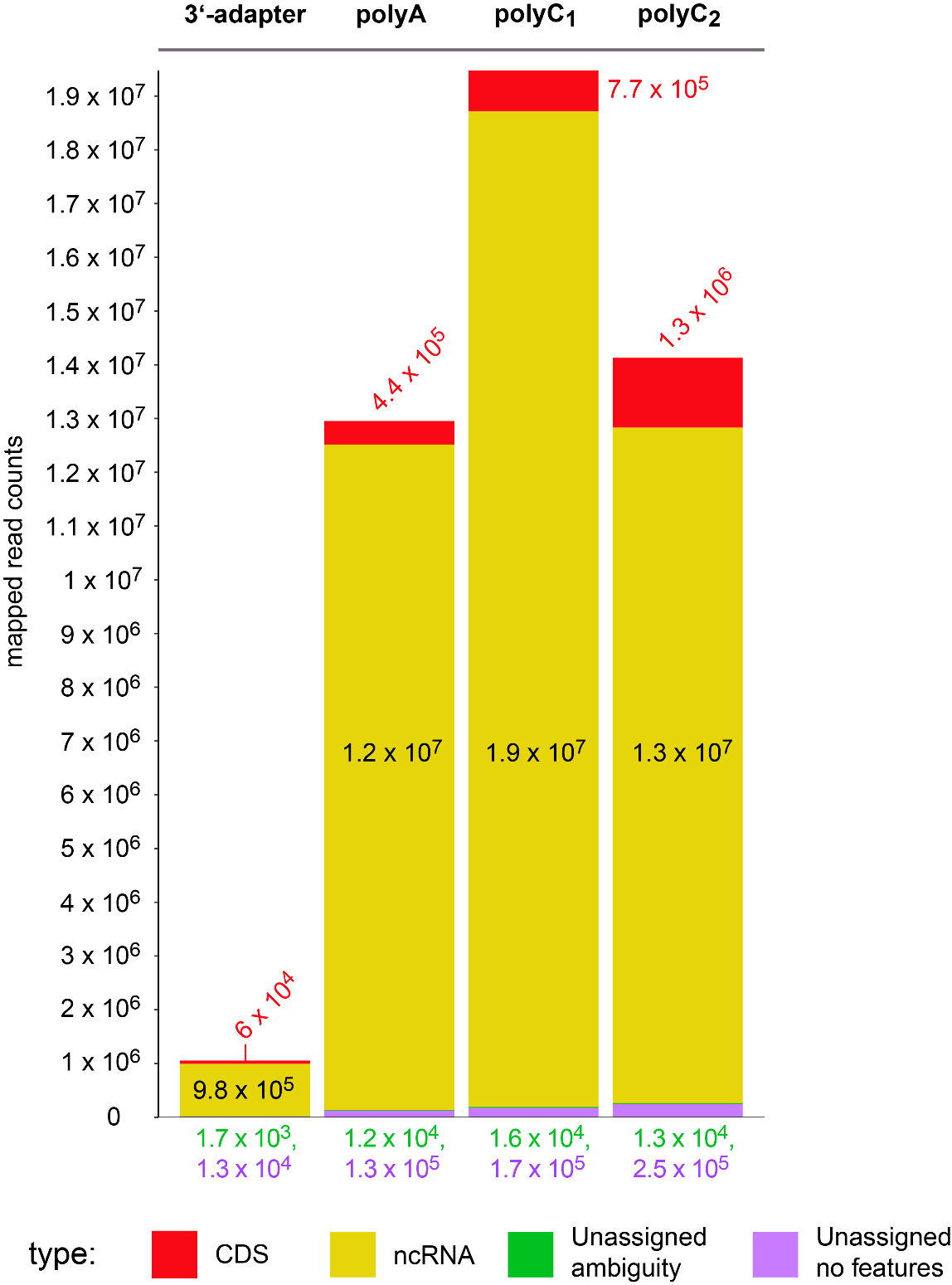
**

**Fig. S3:** Distribution of RNA classes in the four RNA-seq libraries. Most reads were fragments derived from non-coding RNAs (tRNAs, rRNAs and other ncRNAs), followed by RNA fragments mapping to mRNA sequences (classified as coding sequences [CDS] if ≥ 80% of the read maps to a CDS; ≤ 20% may map to UTRs or intergenic regions of polycistronic transcripts). Minor fractions are alignments of reads with genomic regions for which no features are annotated (labeled as 'Unassigned no features'), as well as reads that align with genomic regions to which two or more features have been assigned, such as overlapping genes (labeled 'Unassigned ambiguity').


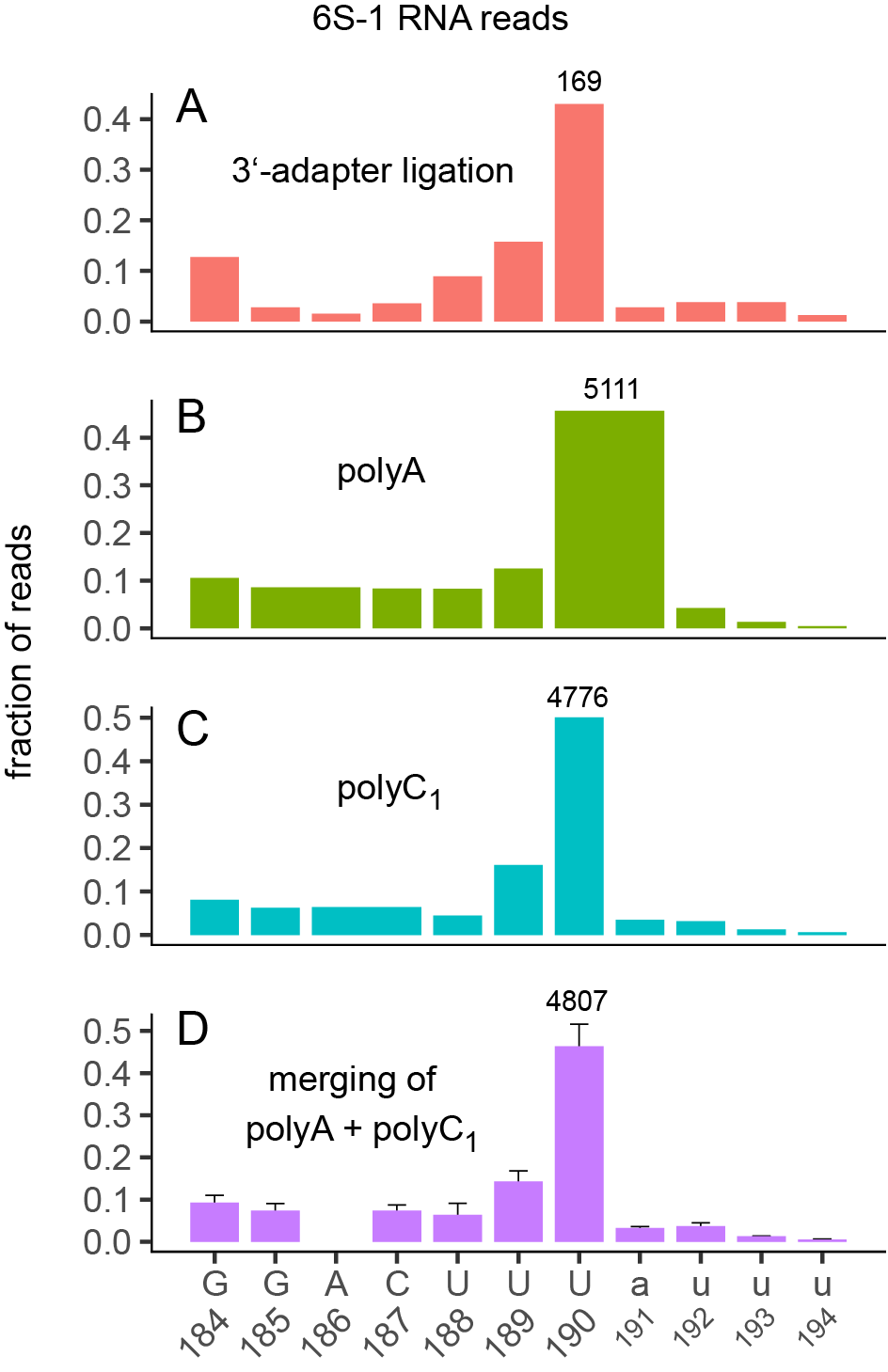


**Fig. S4:** 3'-end distribution of *B. subtilis* 6S-1 RNA reads in the libraries constructed via (**A**) 3'-adapter ligation, (**B**) polyA and (**C**) polyC tailing. Here, only the data of the polyC_1_ library were used, as the protocol was the same as that used for the polyA-tailing library. (**D**) Numerical fit of read lengths by merging the read profiles for the polyA library with that of the polyC_1_ library; for details, see Material and methods and Fig. 5 of the main text, as well as the Supplementary Tables that specify the modus operandi of the algorithm. The 3'-end of mature 6S-1 RNA was previously mapped to position 190 (Suzuma et al., 2002; Wiegard et al., 2023); 3'-precursor nucleotides 191-194 are indicated by lower-case letters. The y-axes depict the fractions of different 3'-ends in reads representing 3'-proximal 6S-1 RNA fragments in the respective libraries. The read number for the most prominent bar is indicated in each profile.

**
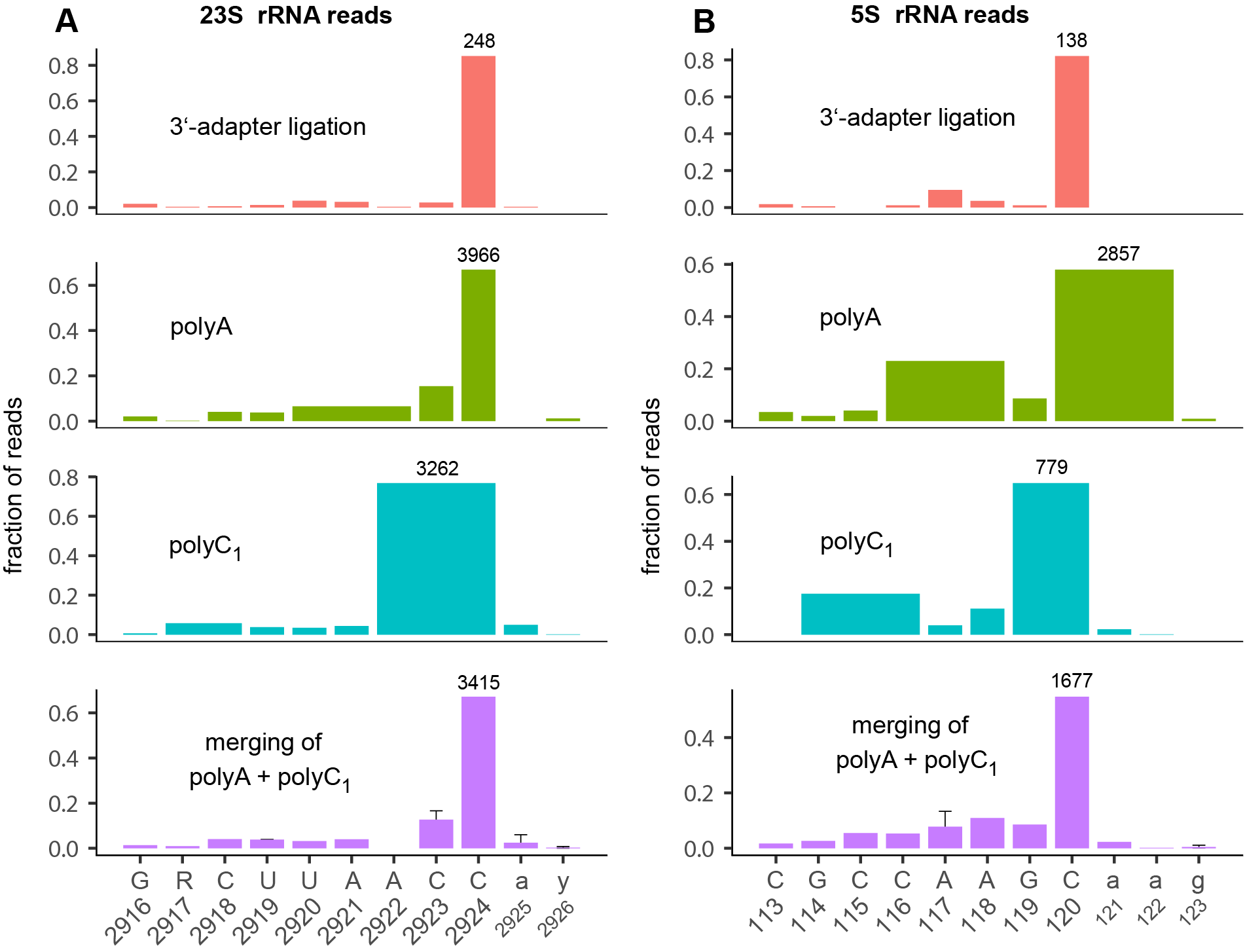
**

**Fig. S5:** 3'-end distribution of *B. subtilis* (**A**) 23S rRNA and (**B**) 5S rRNA in the libraries constructed via 3'-adapter ligation, polyA and polyC tailing. The numerical fit of read lengths by merging the read profiles for the polyA library with that of the polyC_1_ library is shown in the graphs at the bottom. The 3'-end of 23S rRNA was previously mapped to position 2924 (panel A; Redko et al., 2008) and that of 5S rRNA to position 120 (panel B; Sogin and Pace, 1974); 3'-precursor nucleotides are indicated by lower-case letters; R (= purine) and y (= pyrimidine) in panel A is due to sequence heterogeneity among the ten 23S rRNA genes in *B. subtilis*. For more information, see legend to Fig. S4 and Supplementary Tables 23S rRNA and 5S rRNA below.


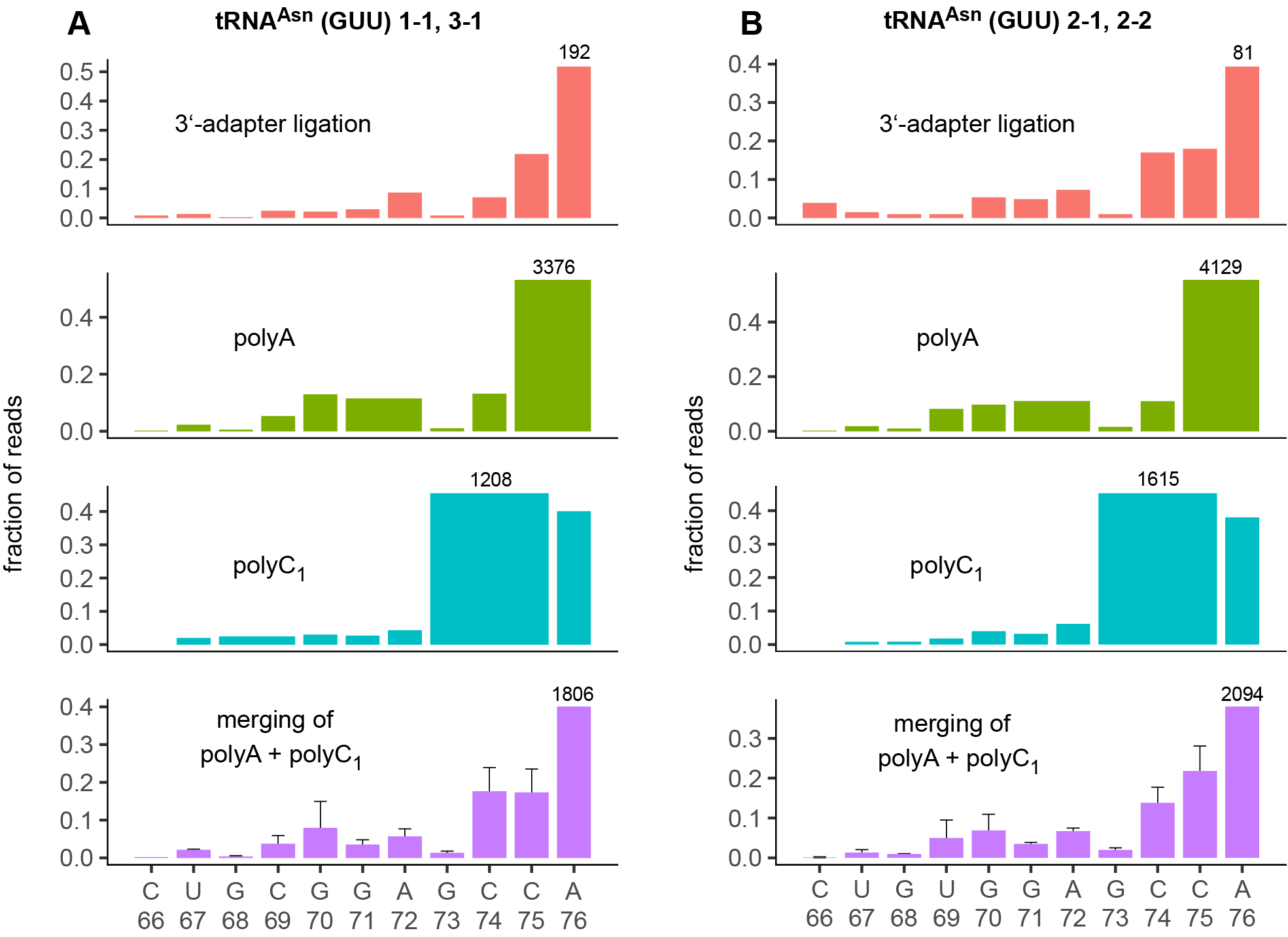


**Fig. S6:** (**A, B**) 3'-end distribution of RNA fragments representing *B. subtilis* tRNAAsn (GUU) isodecoders that differ in sequence at position 69 (nt numbering according to the universal tRNA numbering rules; [Tamaki et al., 2018]), based on libraries constructed via 3'-adapter ligation, polyA and polyC tailing. The numerical fit of read lengths by merging the read profiles for the polyA library with that of the polyC_1_ library is shown in the graphs at the bottom. For more information, see legend to Fig. S4.

**
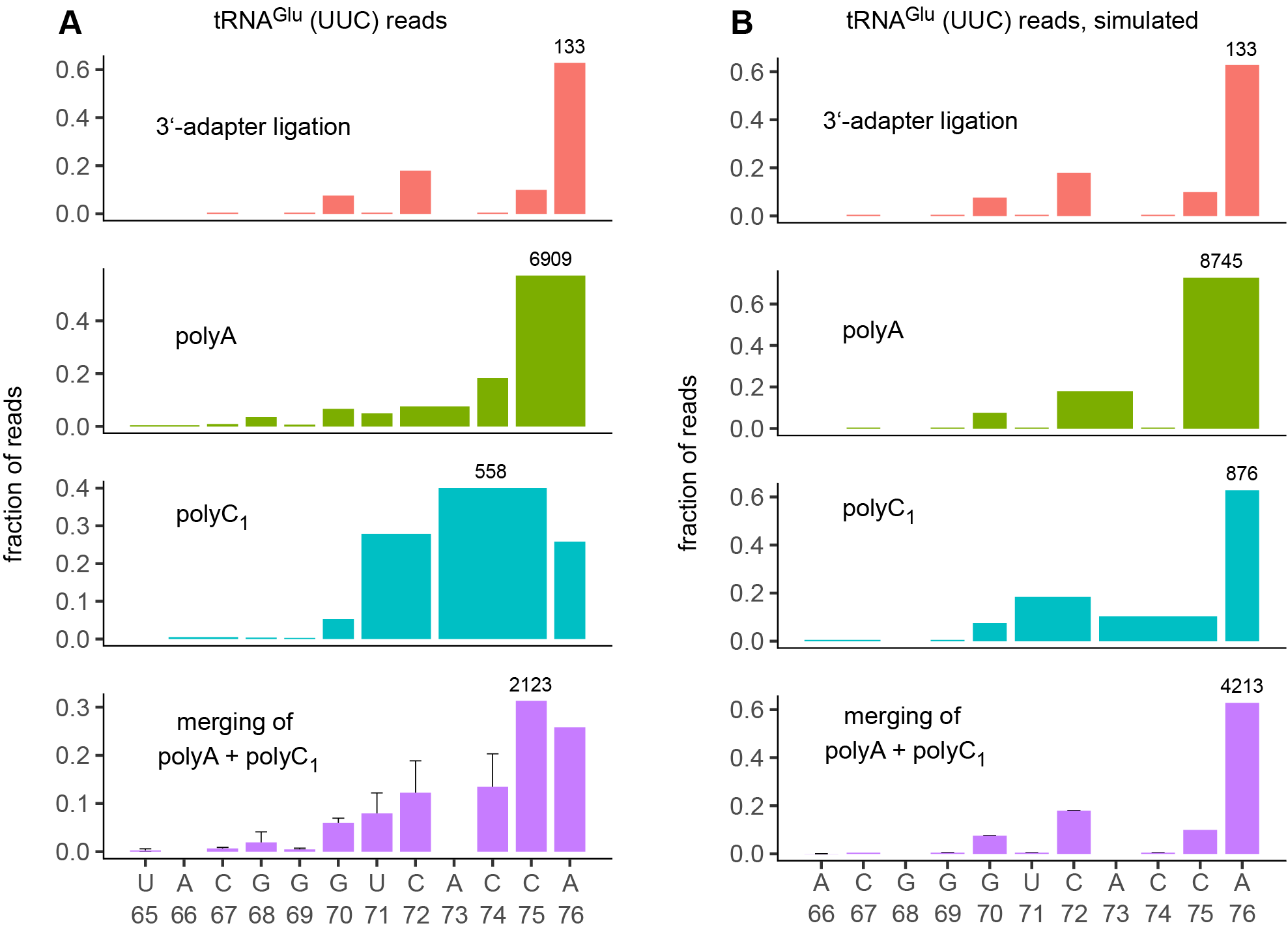
**

**Fig. S7:** (**A**) 3'-end distribution of *B. subtilis* tRNAGlu (UUC), based on libraries constructed via 3'-adapter ligation, polyA and polyC tailing. The numerical fit of read lengths by merging the read profiles for the polyA library with that of the polyC_1_ library is shown in the graph at the bottom. For more information, see legend to Fig. S4 and ‘Supplementary Tables tRNA-Glu’ below. (**B**) Simulated polyA/C-libraries for tRNAGlu (UUC) under the arbitrary assumption that the 3'-adapter libraries conveyed the correct picture. For the simulation approach, see Materials and methods of the main text and ‘Supplementary Tables tRNA-Glu simulated’ below.

**
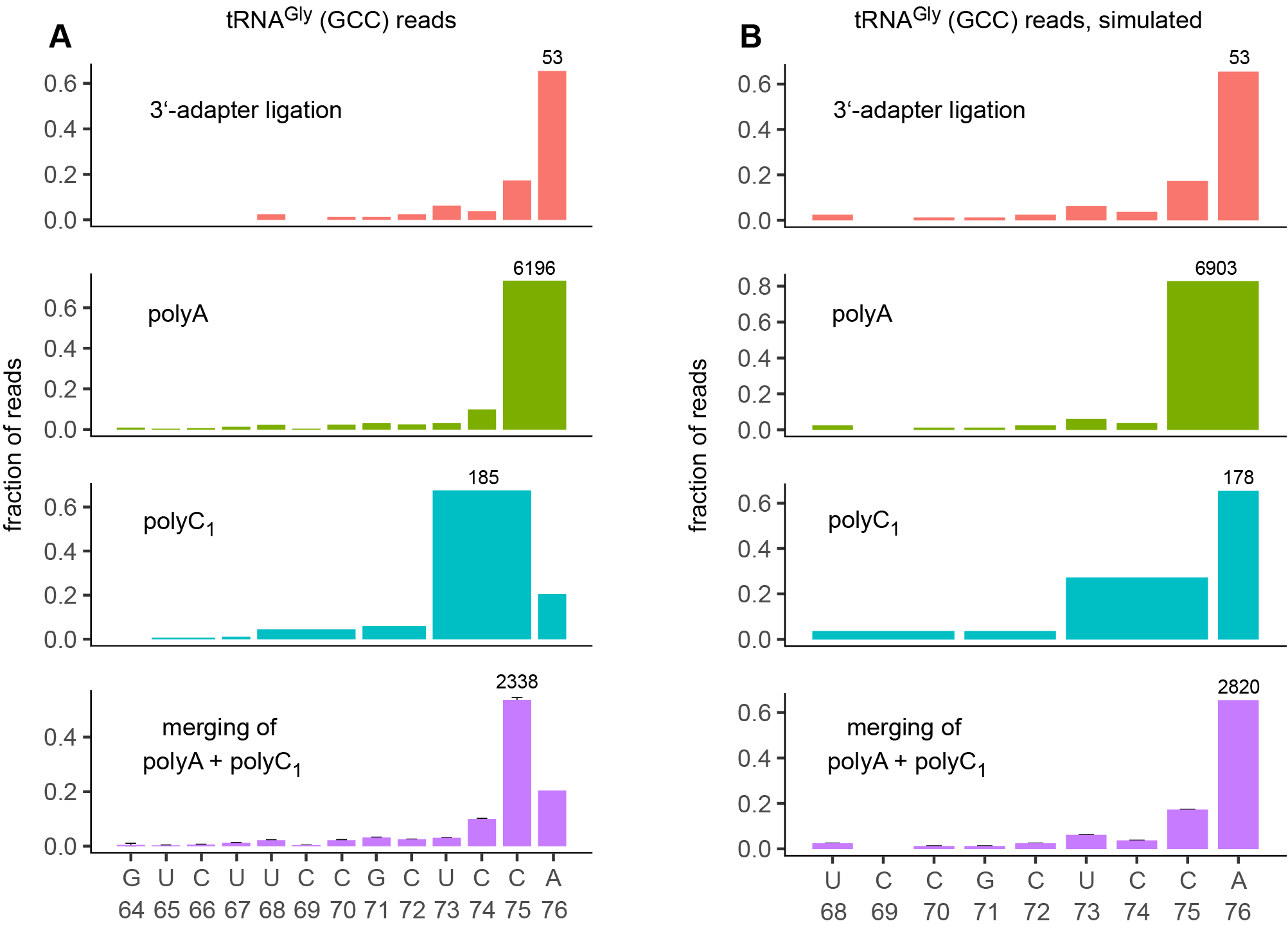
**

**Fig. S8:** (**A**) 3'-end distribution of *B. subtilis* tRNAGly (GCC), based on libraries constructed via 3'-adapter ligation, polyA and polyC tailing. The numerical fit of read lengths by merging the read profiles for the polyA library with that of the polyC_1_ library is shown in the graph at the bottom. For more information, see legend to Fig. S4. (**B**) Simulated polyA/C-libraries for tRNAGly (GCC) under the arbitrary assumption that the 3'-adapter libraries conveyed the correct picture; see Fig. S7B for more information.

**Supplementary Tables and Calculations**

**Supplementary Tables pRNA. Calculations underlying Fig. 4 and 5**

**Merging of polyA and polyC1 libraries**

experimental polyA library pRNA, 5'-_1_GUUCGGUCAAAACUA_15_

|  | read counts  (input) | position | fraction of  reads (*f*) | fraction  × 1000 (*f_1000_*)  (rounded) | merged *f_1000_* | merged read counts  (output) |
| --- | --- | --- | --- | --- | --- | --- |
|  | 4 | G5 | 0.00688468 | 6.88 | 3.44 | 3.63 |
|  | 2 | G6 | 0.00344234 | 3.44 | 2.7 | 2.85 |
|  | 4 | U7 | 0.00688468 | 6.88 | 7.32 | 7.73 |
|  | 488 | C8  A9  A10  A11  A12 | 0.83993115 | 839.93 | 116.09  324.84  240.52  165.36  0 | 122.53  342.87  253.87  174.54  0 |
|  | 48 | C13 | 0.082616179 | 82.62 | 73.34 | 77.41 |
|  | 35 | U14  A15 | 0.06024096 | 60.24 | 65.2  1.2 | 68.82  1.27 |
| Σ | 581 |  | 1 | 999.99 | 1000.01 | 1055.52 |

experimental polyC1 library pRNA, 5'-_1_GUUCGGUCAAAACUA_15_

|  | read counts (input) | position | fraction of  reads (*f*) | fraction  × 1000 (*f_1000_*)  (rounded) | merged *f_1000_* | merged read counts  (output) |
| --- | --- | --- | --- | --- | --- | --- |
|  | 0 | G5 | 0 | 0 | 3.44 | 3.63 |
|  | 3 | G6 | 0.00196078 | 1.96 | 2.7 | 2.85 |
|  | 200 | U7  C8 | 0.13071895 | 130.72 | 7.32  116.09 | 7.73  122.53 |
|  | 497 | A9 | 0.32483660 | 324.84 | 324.84 | 342.87 |
|  | 368 | A10 | 0.240522876 | 240.52 | 240.52 | 253.87 |
|  | 253 | A11 | 0.165359477 | 165.36 | 165.36 | 174.54 |
|  | 98 | A12  C13 | 0.0640522876 | 64.05 | 0  73.34 | 0  77.41 |
|  | 109 | U14 | 0.07124183 | 71.24 | 65.2 | 68.82 |
|  | 2 | A15 | 0.0013071895 | 1.31 | 1.2 | 1.27 |
| Σ | 1530 |  | 1 | 1000 | 1000.01 | 1055.52 |

(581 +1530)/2 = 1055.5


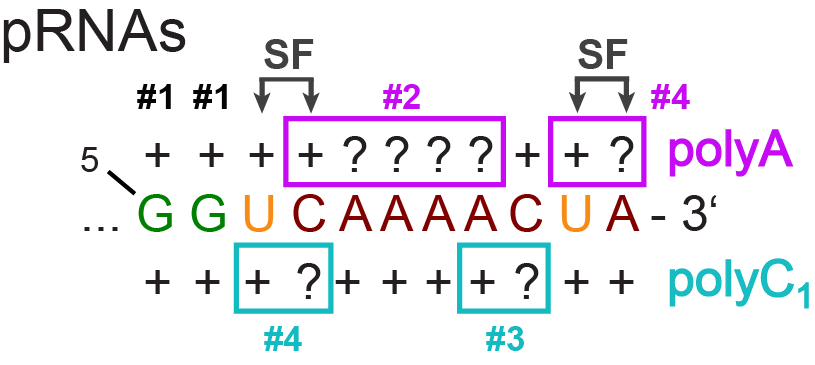
**Calculation of merged values using a pocket calculator**

Position A15/U14 case #4

- A15 unambiguous in polyC1: *f_1000_* = 1.31
- total at U14/A15 in polyA: *f_1000_* = 60.24
- U14 unambiguous in polyC1: *f_1000_* = 71.24
- calculate value for A15 in polyA with scaling factor (SF): 1.31 × 60.24/(71.24 + 1.31) = 1.09
- calculate mean value for A15: (1.31 + 1.09)/2 = 1.2 ± 0.16
- calculate *f_1000_* for U14 in polyA: 71.24 × 60.24/(71.24 + 1.31) = 59.15
- calculate merged mean value for U14: (71.24 + 59.15)/2 = 65.2 ± 8.55

Position C13/A12 case #3

- C13 unambiguous in polyA: *f_1000_* = 82.62
- total at A12/C13 in polyC1: *f_1000_* = 64.05
- negative value for A12 because the total polyC counts at A12/C13 are less than the total polyA counts at C13: 64.05 – 82.62 = –18,57 🡪 0 counts at A12
- calculate mean value for C13: (64.05 + 82.62)/2 = 73.34 ± 13.13

Positions A12/A11/A10/A9/C8 case #2

- A12 = 0 from previous step
- assign polyA counts at C8 to A11 to C8, *f_1000_* = 839.93 (totalA)
- assign counts to A11, A10 and A9 based on polyC1 library: A11, *f_1000_* = 165.36; A10, *f_1000_* = 240.52; A9, *f_1000_* = 324.84
- to obtain counts for C8, subtract the sum of 165.36 + 240.52 + 324.84 = 730.72 (totalC) from totalA: 839.93 – 730.72 = 109.21

Position C8/U7 case #4

- U7 unambiguous in polyA: *f_1000_* = 6.88
- totalC at U7/C8: *f_1000_* = 130.72
- *f_1000_* for C8 from previous step: 109.21
- totalA at U7/C8 = 109.21 + 6.88 = 116.09
- calculate value for U7 in polyC1 with scaling factor (SF): 6.88 × 130.72/116.09) = 7.75
- calculate merged mean value for U7: (6.88 + 7.75)/2 = 7.32 ± 0.62
- correct C8 counts in polyA with SF: *f_1000_* = 109.21 × 130.72/116.09 = 122.97
- calculate merged mean value for C8: (122.97 + 109.21)/2 = 116.09 ± 9.73

Position G6 case #1

- calculate result at position G6 = (3.44 + 1.96 ) / 2 = 2.7 ± 1.05

Position G5 case #1

- calculate result at position G5 = (6.88 + 0) / 2 = 3.44 ± 4.86

Position C4, no read counts

The values for 'merged mean *f_1000_*' are finally divided by 1000 and are multiplied with the mean of the sum of read counts from both libraries, (581 +1530)/2 = 1055.5. As an example, the merged read counts for A9 are 0.32483660 × 1055.5 = 342.87 343 (see right columns in the two tables above).

**Merging of polyA and polyC2 libraries**

experimental polyA library pRNA, 5'-_1_GUUCGGUCAAAACUA_15_

|  | read counts (input) | position | fraction of  reads (*f*) | fraction  × 1000 (*f_1000_*)  (rounded) | merged *f_1000_* | merged read counts  (output) |
| --- | --- | --- | --- | --- | --- | --- |
|  | 4 | G5 | 0.00688468 | 6.88 | 3.51 | 13.85 |
|  | 2 | G6 | 0.00344234 | 3.44 | 2.34 | 9.23 |
|  | 4 | U7 | 0.00688468 | 6.88 | 6.38 | 25.18 |
|  | 488 | C8  A9  A10  A11  A12 | 0.83993115 | 839.93 | 127.67  331.28  203.94  126.66  40.35 | 503.79  1307.23  804.75  499.8  159.22 |
|  | 48 | C13 | 0.082616179 | 82.62 | 82.62 | 326.02 |
|  | 35 | U14  A15 | 0.06024096 | 60.24 | 73.77  1.5 | 291.1  5.92 |
| Σ | 581 |  | 1 | 999.99 | 1000.02 | 3946.09 |

experimental polyC_2_ library pRNA, 5'-_1_GUUCGGUCAAAACUA_15_

|  | read counts (input) | position | fraction of  reads (*f*) | fraction  × 1000 (*f_1000_*)  (rounded) | merged *f_1000_* | merged read counts  (output) |
| --- | --- | --- | --- | --- | --- | --- |
|  | 1 | G5 | 0.000136780194 | 0.14 | 3.51 | 13.85 |
|  | 9 | G6 | 0.001231021748 | 1.23 | 2.34 | 9.23 |
|  | 903 | U7  C8 | 0.123512515388 | 123.51 | 6.38  127.67 | 25.18  503.79 |
|  | 2422 | A9 | 0.3312816304199 | 331.28 | 331.28 | 1307.23 |
|  | 1491 | A10 | 0.20393926959376 | 203.94 | 203.94 | 804.75 |
|  | 926 | A11 | 0.126658459855 | 126.66 | 126.66 | 499.8 |
|  | 899 | A12  C13 | 0.12296539461086 | 122.97 | 40.35  82.62 | 159.22  326.02 |
|  | 647 | U14 | 0.0884967856654 | 88.5 | 73.77 | 291.1 |
|  | 13 | A15 | 0.00177814252496 | 1.8 | 1.5 | 5.92 |
| Σ | 7311 |  | 1 | 1000.03 | 1000.02 | 3946.09 |

(581 +7311)/2 = 3946

**
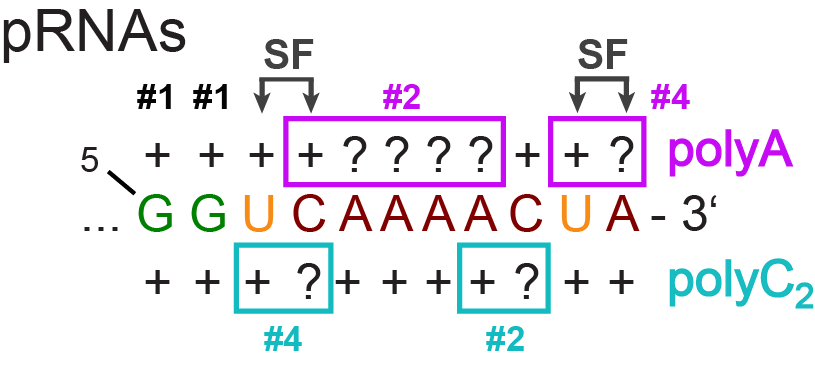
Calculation of merged values using a pocket calculator**

Position A15/U14 case #4

- A15 unambiguous in polyC2: *f_1000_* = 1.8
- total at U14/A15 in polyA: *f_1000_* = 60.24
- U14 unambiguous in polyC2: *f_1000_* = 88.5
- calculate value for A15 in polyA with scaling factor (SF): 1.8 × 60.24/(88.5 + 1.8) = 1.2
- calculate mean value for A15: (1.8 + 1.2)/2 = 1.5 ± 0.42
- calculate *f_1000_* for U14 in polyA: 88.5 × 60.24/(88.5 + 1.8) = 59.04
- calculate merged mean value for U14: (88.5 + 59.04)/2 = 73.77 ± 20.83

Position C13/A12 case #2

- C13 unambiguous in polyA: *f_1000_* = 82.62
- total at A12/C13 in polyC2: *f_1000_* = 122.97
- calculate value for A12 in polyC: *f_1000_* = 122.97 – 82.62 = 40.35

Positions A12/A11/A10/A9/C8 case #2

- A12 from previous step: *f_1000_* = 40.35
- assign polyA counts at C8 to A12 to C8, *f_1000_* = 839.93 (totalA)
- assign counts to A12, A11, A10 and A9 based on polyC2 library: A12, *f_1000_* = 40.35; A11, *f_1000_* = 126.66; A10, *f_1000_* = 203.94; A9, *f_1000_* = 331.28
- to obtain counts for C8, subtract the sum of 40.35 + 126.66 + 203.94 + 331.28 = 702.23 (totalC) from totalA: 839.93 – 702.23 = 137.7; *f_1000_* = 137.7 for C8 in polyA

Position C8/U7 case #4

- U7 unambiguous in polyA: *f_1000_* = 6.88
- totalC at U7/C8: *f_1000_* = 123.51
- *f_1000_* for C8 in polyA from previous step: 137.7
- totalA at U7/C8 = 137.7+ 6.88 = 144.58
- calculate value for U7 in polyC2 with scaling factor (SF): 6.88 × 123.51/144.58) = 5.88
- calculate merged mean value for U7: (6.88 + 5.88)/2 = 6.38 ± 0.71
- correct C8 counts in polyA with SF: *f_1000_* = 137.7 × 123.51/144.58 = 117.63
- calculate merged mean value for C8: (117.63 + 137.7)/2 = 127.67 ± 14.19

Position G6 case #1

- calculate result at position G6 = (3.44 + 1.23) / 2 = 2.34 ± 1.56

Position G5 case #1

- calculate result at position G5 = (6.88 + 0.14) / 2 = 3.51 ± 4.77

The values for 'merged mean *f_1000_*' are finally divided by 1000 and are multiplied with the mean of the sum of read counts from both libraries, (581 +7311)/2 = 3946. As an example, the merged read counts for A9 are 0.33128 × 3946 = 1307.23 1307 (right columns in the polyA/polyC2 tables above).

**Corresponding algorithm protocol for the merging of polyA and polyC1 libraries**

Table of raw read counts:

pos seq A C G T

1 G 0 0 0 0

2 T 0 0 0 0

3 T 0 0 0 0

4 C 0 0 0 0

5 G 4 0 0 0

6 G 2 3 0 0

7 T 4 200 0 0

8 C 488 0 0 0

9 A 0 497 0 0

10 A 0 368 0 0

11 A 0 253 0 0

12 A 0 98 0 0

13 C 48 0 0 0

14 T 35 109 0 0

15 A 0 2 0 0

total raw read counts of A is 581

total raw read counts of C is 1530

total raw read counts of G is 0

total raw read counts of T is 0

Table of factors (relative to total raw read counts):

pos seq A C G T

1 G 0 0 0 0

2 T 0 0 0 0

3 T 0 0 0 0

4 C 0 0 0 0

5 G 0.00688468158347676 0 0 0

6 G 0.00344234079173838 0.00196078431372549 0 0

7 T 0.00688468158347676 0.130718954248366 0 0

8 C 0.839931153184165 0 0 0

9 A 0 0.32483660130719 0 0

10 A 0 0.240522875816993 0 0

11 A 0 0.165359477124183 0 0

12 A 0 0.0640522875816993 0 0

13 C 0.0826161790017212 0 0 0

14 T 0.0602409638554217 0.0712418300653595 0 0

15 A 0 0.00130718954248366 0 0

Table of factors * 1000:

pos seq A C G T

1 G 0 0 0 0

2 T 0 0 0 0

3 T 0 0 0 0

4 C 0 0 0 0

5 G 6.88 0 0 0

6 G 3.44 1.96 0 0

7 T 6.88 130.71 0 0

8 C 839.93 0 0 0

9 A 0 324.83 0 0

10 A 0 240.52 0 0

11 A 0 165.35 0 0

12 A 0 64.05 0 0

13 C 82.61 0 0 0

14 T 60.24 71.24 0 0

15 A 0 1.3 0 0

Position A15 case #4

- define the factor (0+60.24) / (1.3+0+0+71.24+0+0) = 0.8303

- calculate result at position 15 = (1.3 + 1.3 * 0.8303 (factor))/2 = 1.19 (SD=0.15)

- correct A15 in library C from 1.3 to mean(1.3, 1.3 * 0.8303 (factor)) = 1.19

Position T14 case #4

- define the factor (0+60.24) / (1.3+0+0+71.24+0+0) = 0.8303

- calculate result at position 14 = (71.24 + 71.24 * 0.8303 (factor))/2 = 65.19 (SD=8.54)

- correct T14 in library A from 60.24 to mean(60.24, 60.24 * 0.8303 (factor)) = 55.13

- correct T14 in library C from 71.24 to mean(71.24, 71.24 * 0.8303 (factor)) = 65.19

Position C13 case #3

- the reference amount 64.05 < 0 (current total) + 82.61 + 0 (current position), this is an inconcordance between the libraries. I will now instead average the information and distribute accordingly.

- calculate result at position 13 = ((64.05-0) + ( 82.61 + 0)/1)/2 = 73.33 (SD=13.12)

- correct A12 in library C from 64.05 to 64.05-73.33 = 0

Position A12 nothing left

- calculate result at position 12 = 0

Position A11 A case #2

- calculate result at position 11 = 165.35 (polyC) (SD=0)

Position A10 A case #2

- calculate result at position 10 = 240.52 (polyC) (SD=0)

Position A9 A case #2

- calculate result at position 9 = 324.83 (polyC) (SD=0)

- correct C8 in library A from 839.93 to 839.93-730.71 = 109.21

Position C8 case #4

- define the factor (0+130.71) / (109.21+0+0+6.88+0+0) = 1.1259

- calculate result at position 8 = (109.21 + 109.21 * 1.1259 (factor))/2 = 116.08 (SD=9.72)

- correct C8 in library A from 109.21 to mean(109.21, 109.21 * 1.1259 (factor)) = 116.08

Position T7 case #4

- define the factor (0+130.71) / (109.21+0+0+6.88+0+0) = 1.1259

- calculate result at position 7 = (6.88 + 6.88 * 1.1259 (factor))/2 = 7.31 (SD=0.61)

- correct T7 in library A from 6.88 to mean(6.88, 6.88 * 1.1259 (factor)) = 7.31

- correct T7 in library C from 130.71 to mean(130.71, 130.71 * 1.1259 (factor)) = 138.95

Position G6 case #1

- calculate result at position 6 = ( 3.44 + 1.96 ) / 2 = 2.7 (SD=1.04)

Position G5 case #1

- calculate result at position 5 = ( 6.88 + 0 ) / 2 = 3.44 (SD=4.86)

Position C4 nothing left

- calculate result at position 4 = 0

Position T3 case #1

- calculate result at position 3 = ( 0 + 0 ) / 2 = 0 (SD=0)

Position T2 case #1

- calculate result at position 2 = ( 0 + 0 ) / 2 = 0 (SD=0)

Position G1 case #1

- calculate result at position 1 = ( 0 + 0 ) / 2 = 0 (SD=0)

average total read count = 1055.5 = 2111/2, multiplying the result by this factor:

final result:

# pos fraction of merged reads merged read counts SD fraction of merged reads SD merged reads nt

1 0 0 0 0 G

2 0 0 0 0 T

3 0 0 0 0 T

4 0 0 0 0 C

5 0.00344234079173838 3.63339070567986 0.00486820503398656 5.13839041337281 G

6 0.00270156255273194 2.85149927440856 0.00104761863231377 1.10576146640719 G

7 0.007318234669764 7.72439669393591 0.000613136654636128 0.647165738968433 T

8 0.116089682714057 122.532660104687 0.00972623083421316 10.266036645512 C

9 0.32483660130719 342.865032679739 0 0 A

10 0.240522875816993 253.871895424837 0 0 A

11 0.165359477124183 174.536928104575 0 0 A

12 0 0 0 0 A

13 0.0733342332917103 77.4042832394002 0.0131266535083082 13.8551827780193 C

14 0.0651986855743057 68.8172126236797 0.0085462968986285 9.02061637650238 T

15 0.00119630615732671 1.26270114905834 0.000156812787130798 0.165515896816557 A

**Corresponding algorithm protocol for the merging of polyA and polyC2 libraries**

Table of raw read counts:

pos seq A C G T

1 G 0 0 0 0

2 T 0 0 0 0

3 T 0 0 0 0

4 C 0 0 0 0

5 G 4 1 0 0

6 G 2 9 0 0

7 T 4 903 0 0

8 C 488 0 0 0

9 A 0 2422 0 0

10 A 0 1491 0 0

11 A 0 926 0 0

12 A 0 899 0 0

13 C 48 0 0 0

14 T 35 647 0 0

15 A 0 13 0 0

total raw read counts of A is 581

total raw read counts of C is 7311

total raw read counts of G is 0

total raw read counts of T is 0

Table of factors (relative to total raw read counts):

pos seq A C G T

1 G 0 0 0 0

2 T 0 0 0 0

3 T 0 0 0 0

4 C 0 0 0 0

5 G 0.00688468158347676 0.000136780194227876 0 0

6 G 0.00344234079173838 0.00123102174805088 0 0

7 T 0.00688468158347676 0.123512515387772 0 0

8 C 0.839931153184165 0 0 0

9 A 0 0.331281630419915 0 0

10 A 0 0.203939269593763 0 0

11 A 0 0.126658459855013 0 0

12 A 0 0.12296539461086 0 0

13 C 0.0826161790017212 0 0 0

14 T 0.0602409638554217 0.0884967856654356 0 0

15 A 0 0.00177814252496239 0 0

Table of factors * 1000:

pos seq A C G T

1 G 0 0 0 0

2 T 0 0 0 0

3 T 0 0 0 0

4 C 0 0 0 0

5 G 6.88 0.13 0 0

6 G 3.44 1.23 0 0

7 T 6.88 123.51 0 0

8 C 839.93 0 0 0

9 A 0 331.28 0 0

10 A 0 203.93 0 0

11 A 0 126.65 0 0

12 A 0 122.96 0 0

13 C 82.61 0 0 0

14 T 60.24 88.49 0 0

15 A 0 1.77 0 0

Position A15 case #4

- define the factor (0+60.24) / (1.77+0+0+88.49+0+0) = 0.6673

- calculate result at position 15 = (1.77 + 1.77 * 0.6673 (factor))/2 = 1.48 (SD=0.41)

- correct A15 in library C from 1.77 to mean(1.77, 1.77 * 0.6673 (factor)) = 1.48

Position T14 case #4

- define the factor (0+60.24) / (1.77+0+0+88.49+0+0) = 0.6673

- calculate result at position 14 = (88.49 + 88.49 * 0.6673 (factor))/2 = 73.77 (SD=20.81)

- correct T14 in library A from 60.24 to mean(60.24, 60.24 * 0.6673 (factor)) = 50.22

- correct T14 in library C from 88.49 to mean(88.49, 88.49 * 0.6673 (factor)) = 73.77

Position C13 C case #2

- calculate result at position 13 = 82.61 (polyA) (SD=0)

- correct A12 in library C from 122.96 to 122.96-82.61 = 40.34

Position A12 A case #2

- calculate result at position 12 = 40.34 (polyC) (SD=0)

Position A11 A case #2

- calculate result at position 11 = 126.65 (polyC) (SD=0)

Position A10 A case #2

- calculate result at position 10 = 203.93 (polyC) (SD=0)

Position A9 A case #2

- calculate result at position 9 = 331.28 (polyC) (SD=0)

- correct C8 in library A from 839.93 to 839.93-702.22 = 137.7

Position C8 case #4

- define the factor (0+123.51) / (137.7+0+0+6.88+0+0) = 0.8542

- calculate result at position 8 = (137.7 + 137.7 * 0.8542 (factor))/2 = 127.66 (SD=14.19)

- correct C8 in library A from 137.7 to mean(137.7, 137.7 * 0.8542 (factor)) = 127.66

Position T7 case #4

- define the factor (0+123.51) / (137.7+0+0+6.88+0+0) = 0.8542

- calculate result at position 7 = (6.88 + 6.88 * 0.8542 (factor))/2 = 6.38 (SD=0.7)

- correct T7 in library A from 6.88 to mean(6.88, 6.88 * 0.8542 (factor)) = 6.38

- correct T7 in library C from 123.51 to mean(123.51, 123.51 * 0.8542 (factor)) = 114.51

Position G6 case #1

- calculate result at position 6 = ( 3.44 + 1.23 ) / 2 = 2.33 (SD=1.56)

Position G5 case #1

- calculate result at position 5 = ( 6.88 + 0.13 ) / 2 = 3.51 (SD=4.77)

Position C4 nothing left

- calculate result at position 4 = 0

Position T3 case #1

- calculate result at position 3 = ( 0 + 0 ) / 2 = 0 (SD=0)

Position T2 case #1

- calculate result at position 2 = ( 0 + 0 ) / 2 = 0 (SD=0)

Position G1 case #1

- calculate result at position 1 = ( 0 + 0 ) / 2 = 0 (SD=0)

average total read count = 3946 = 7892/2, multiplying the result by this factor:

final result:

# pos fraction of merged reads merged read counts SD fraction of merged reads SD merged reads nt

1 0 0 0 0 G

2 0 0 0 0 T

3 0 0 0 0 T

4 0 0 0 0 C

5 0.00351073088885232 13.8533440874113 0.00477148683111601 18.8282870355838 G

6 0.00233668126989463 9.22054429100422 0.00156363869115838 6.17011827531098 G

7 0.00638293301333464 25.1870536706185 0.000709579632796301 2.8000012310142 T

8 0.127666954325457 503.773801768254 0.014192514692106 56.0036629750501 C

9 0.331281630419915 1307.23731363699 0 0 A

10 0.203939269593763 804.744357816988 0 0 A

11 0.126658459855013 499.794282587881 0 0 A

12 0.0403492156091392 159.218004793663 0 0 A

13 0.0826161790017212 326.003442340792 0 0 C

14 0.0737755925406404 291.118488165367 0.020818910971399 82.1514226931403 T

15 0.00148235348226944 5.8493668410352 0.000418308875777723 1.65064682381889 A

**Comments:** The algorithm is designed to be able to do the merging of four libraries constructed by 3’ tailing with A, C, G and U, respectively, explaining why the ‘Table of raw read counts’ lists columns for all 4 nucleotides; the algorithm works with T instead of U residues, as RNA-seq readout is on the cDNA level. In the case of the 6S-1 pRNA example, the position numbering is identical to the numbering of 6S-1 pRNA nucleotides; in other cases (e.g. 6S-1 RNA, tRNAs, 23S and 5S rRNAs), the consecutive numbering of positions in the protocol above has to be correlated with the sequence numbering for the respective RNA. In the calculation lines (see examples highlighted in yellow), *f*1000 sums include 0 values as place holders for additional libraries (polyG, polyU or replicates of polyA or polyC libraries). Abbreviations: nt, nucleotide; SD, standard deviation; pos, position; seq, sequence.

**Supplementary Tables 23S rRNA. Calculations underlying Fig. S5A**

**3’ ends of 23S rRNAs transcripts in *B. subtilis***

2916 2925

│ │

*rrnO:* … gacUUaacc a u a u u u u u-3’

2925

│

*rrnA:* … gacUUaacc a c a u u u u g-3’

2925

│

*rrnJ:* … gacUUaacc a u a u u u u u-3’

2925

│

*rrnG:* … gacUUaacc a u a u u u u u-3’

2925

│

*rrnW:* … gacUUaacc a u a u u u u u-3’

2923

│

*rrnI:* … gacUUaacc a u a u u u u u-3’

2921

│

*rrnH:* … gacUUaacc a u a u u u u u-3’

2925

│

*rrnE:* … gacUUaacc a u a u u u u u-3’

2925

│

*rrnD:* … gGcUUaacc a u a u u u u u-3’

2925

│

*rrnB:* … GACUUaacc a u a u u u u u-3’-3’

The 10 rRNA operons of *B. subtilis* are almost sequence-identical in their 3’ end regions. Only the *rrnD* operon carries an A to G substitution (marked in red) at position 2917, which was ignored for simplicity’s sake in the following analysis; the same pertains the U to C exchange at position 2926 of the *rrnA* operon. The *rrnI* and *rrnH* operons encode 23S rRNAs a few nucleotides shorter than those encoded by the other 8 *rrn* operons, thus their genomic numbering slightly differs (red numbers above the sequence). We applied the numbering of the other eight 23S rRNA transcripts also to these two.

**Merging of experimental polyA and polyC1 libraries for 23S rRNA**

experimental polyA library 23S rRNA

|  | read counts (input) | position | fraction of  reads (*f*) | fraction  × 1000, (*f_1000_*)  (rounded) | merged *f_1000_* | merged read counts (output) |
| --- | --- | --- | --- | --- | --- | --- |
|  | 121 | G2916  A2917 | 0.0204426423382328 | 20.44 | 6.32  15.87 | 32.14  80.71 |
|  | 241 | C2918 | 0.0407163372191249 | 40.72 | 40.72 | 207.1 |
|  | 224 | U2919 | 0.0378442304443318 | 37.84 | 38.09 | 193.73 |
|  | 385 | U2920  A2921  A2922 | 0.0650447710761953 | 65.04 | 31.86  39.82  0 | 162.04  202.52  0 |
|  | 915 | C2923 | 0.154586923466802 | 154.59 | 150.45 | 765.19 |
|  | 3966 | C2924  A2925 | 0.670045615813482 | 670.05 | 620.67  49.38 | 3156.73  251.13 |
|  | 67 | U2926  A2927 | 0.0113194796418314 | 11.32 | 3.42  3.42 | 17.93  17.93 |
| Σ | 5919 |  | 1 | 1000 | 1000.02 | 5087.15 |

experimental polyC_1_ library 23S rRNA

|  | read counts (input) | position | fraction of  reads | fraction × 1000 (*f_1000_*) (rounded) | merged *f_1000_* | merged read counts (output) |
| --- | --- | --- | --- | --- | --- | --- |
|  | 29 | G2916 | 0.00681871620032918 | 6.82 | 6.32 | 32.14 |
|  | 246 | A2917  C2918 | 0.0578415236303786 | 57.84 | 15.87  40.72 | 80.71  207.1 |
|  | 163 | U2919 | 0.0383258876087468 | 38.33 | 38.09 | 193.73 |
|  | 148 | U2920 | 0.0347989654361627 | 34.8 | 31.86 | 162.04 |
|  | 185 | A2921 | 0.0434987067952034 | 43.5 | 39.82 | 202.52 |
|  | 3262 | A2922  C2923  C2924 | 0.766988008464613 | 766.99 | 0  150.45  620.67 | 0  765.19  3156.73 |
|  | 210 | A2925 | 0.0493769104161768 | 49.38 | 49.38 | 251.13 |
|  | 5 | U2926 | 0.00117564072419469 | 1.18 | 3.42 | 17.93 |
|  | 5 | A2927 | 0,00117564072419469 | 1.18 | 3.42 | 17.93 |
| Σ | 4253 |  | 1 | 1000.02 | 1000.02 | 5087.15 |

5919 + 4253 = 10172/2 = 5086


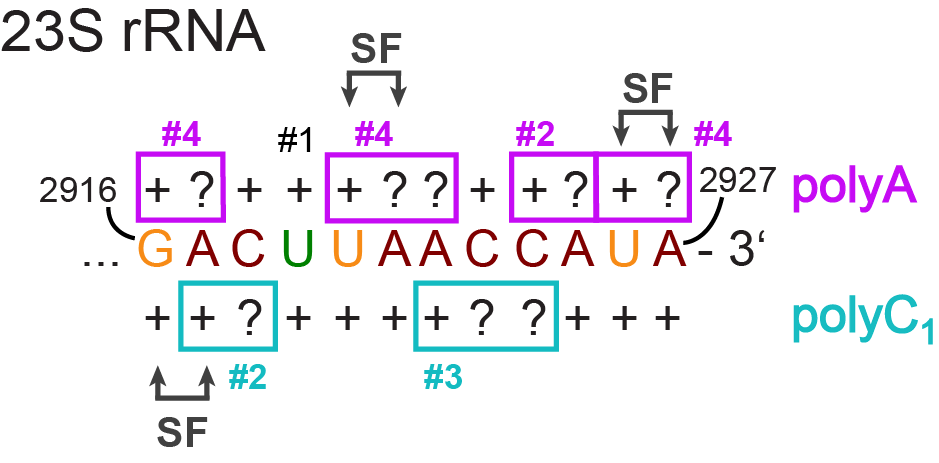
**Calculation of merged values using a pocket calculator**

Position A2927/U2926 case #4

- A2927 unambiguous in polyC_1_: *f_1000_* = 1.18
- U2926 unambiguous in polyC_1_: *f_1000_* = 1.18
- total at U2926/A2927 in polyA: *f_1000_* = 11.32
- calculate value for A2927 in polyC1 with scaling factor (SF): *f_1000_* = 1.18 × 11.32/(1.18 +1.18) = 5.66
- calculate value for U2926 in polyC1 with scaling factor (SF): *f_1000_* = 1.18 × 11.32/(1.18 +1.18) = 5.66
- calculate merged mean value for A2927: *f_1000_* = (1.18 + 5.66)/2 = 3.42 ± 3.17
- calculate merged mean value for U2926: *f_1000_* = (1.18 + 5.66)/2 = 3.42 ± 3.17

Position A2925/C2924 case #2

- A2925 unambiguous case in polyC1: *f_1000_* = 49.38
- totalA at C2924/A2925: *f_1000_* = 670.05
- calculate C2924 in polyA: *f_1000_* = 670.05– 49.38 = 620.67

Position C2923/A2922 case #3

- totalC at A2922/C2923/C2924: *f_1000_* = 766.99
- C2923 unambiguous in polyA: *f_1000_* = 154.59
- calculate value for A2922: *f_1000_* = 766.99– 620.67 – 154.59 = – 8.27 🡪 *f_1000_* = 0 for A2922
- correct *f_1000_* for C2923: 766.98-620.66 = 146.32 🡪 calculate mean (154.58 + 146.32)/2 = 150.45 ± 5.84

Position A2921/U2920 case #4

- A2921 unambiguous in polyC_1_: *f_1000_* = 43.5
- U2920 unambiguous in polyC_1_: *f_1000_* = 34.8
- *f_1000_* = 0 for A2922 from previous step
- totalA at U2920/A2921: *f_1000_* = 65.04
- totalC at U2920/A2921: *f_1000_* = 43.5 + 34.8 = 78.3
- calculate value for A2921 in polyC_1_ with scaling factor (SF): *f_1000_* = 43.5 × 65.04/78.3= 36.13
- calculate value for U2920 in polyC_1_ with scaling factor (SF): *f_1000_* = 34.8 × 65.04/78.3 = 28.91
- calculate merged mean value for A2921: *f_1000_* = (43.5 + 36.13)/2 = 39.82 ± 5.21
- calculate merged mean value for U2920: *f_1000_* = (34.8 + 28.91)/2 = 31.86 ± 4.16

Position U2919 case #1

- calculate mean value in polyA and polyC_1_ for U2919: *f_1000_* = (37.84 + 38.33)/2 = 38.09 ± 0.35

Position C2918 case #2

- C2918 unambiguous in polyA: *f_1000_* = 40.72
- totalC at C2918/A2917: *f_1000_* = 57.84
- calculate *f_1000_* for A2917 in polyC_1_: 57.84 – 40.72 = 17.12
- correct value for A2917 in polyC_1_ with scaling factor (SF): *f_1000_* = 17.12 × 20.44/(6.82 + 17.12) = 14.62
- calculate merged mean value for A2917: *f_1000_* = (17.12 + 14.62)/2 = 15.87 ± 1.77

Position G2916/A2917 case #4

- G2916 unambiguous in poly C_1_: *f_1000_* = 6.82
- A2917 in polyC_1_ from previous step: *f_1000_* = 17.12
- totalA at G2916/A2917: *f_1000_* = 20.44
- calculate *f_1000_* for G2916 in polyC_1_ with scaling factor (SF): *f_1000_* = 6.82 × 20.44/(6.82 + 17.12) = 5.82
- calculate merged mean value for G2916: *f_1000_* = (6.82 + 5.82)/2 = 6.32 ± 0.71

The values for for 'merged mean *f_1000_*' are finally divided by 1000 and are multiplied with the mean of the sum of read counts from both libraries 5919 + 4253 = 10172/2 = 5086 (see right column in the tables above).

**Supplementary Tables 5S rRNA. Calculations underlying Fig. S5B**

**3’ ends of 5S rRNAs transcripts in *B. subtilis***

116

│

*rrnO:* … GCCAAGC a a g c u-3’

116

│

*rrnA:* … GCCAAGC a a g c u-3’

116

│

*rrnJ:* … GCCAAGC a a u u -3’

116

│

*rrnW:* … GCCAAGC a a g c u -3’

116

│

*rrnI:* … GCCAAGC a g a u u -3’

116

│

*rrnH:* … GCCAAGC a a u u -3’

116

│

*rrnG:* … GCCAAGC a a g c u-3’

116

│

*rrnE:* … CGCCAAGC a u u a c u-3’

116

│

*rrnD:* … GCCAGGC a g a c u-3’

116

│

*rrnB:* … GCCAAGC a a u u -3’

Nine of the ten 5S rRNA transcripts have an A at position 114 (except for a G in the *rrnD* operon, see above, marked in red). Likewise, seven of the ten 5S rRNA transcripts have an A at position 118, two a G and one a U. For simplicity’s sake, the nucleotide identities at positions 114 and 118 were treated for all ten 5S rRNA genes as A in the following analysis.

**Merging of experimental polyA and polyC1 libraries for 5S rRNA**

experimental polyA library 5S rRNA

|  | read  counts (input) | position | fraction of  reads (*f*) | fraction  × 1000, (*f_1000_*)  (rounded) | merged *f_1000_* | merged read counts  (output) |
| --- | --- | --- | --- | --- | --- | --- |
|  | 96 | G110 | 0.0203691915977085 | 20.37 | 21.93 | 64.85 |
|  | 199 | C111 | 0.0422236367494165 | 42.22 | 45.45 | 134.40 |
|  | 1134 | C112  A113  A114 | 0.240611075747931 | 240.61 | 95.88  39.97  111.57 | 283.52  118.19  329.91 |
|  | 427 | G115 | 0.0906004667939741 | 90.6 | 89.04 | 263.29 |
|  | 2857 | C116  A117  A118 | 0.60619562911097 | 606.2 | 571.19  23.31  1.67 | 1689.01  68.93  4.94 |
| Σ | 4713 |  | 1 | 1000 | 1000.01 |  |

experimental polyC1 library 5S rRNA

|  | read  counts (input) | position | fraction of  reads (*f*) | fraction  × 1000, (*f_1000_*)  (rounded) | merged *f_1000_* | merged read counts  (output) |
| --- | --- | --- | --- | --- | --- | --- |
|  | 210 | G110  C111  C112 | 0.174854288093256 | 174.85 | 21.93  45.45  95.88 | 64.85  134.40  283.52 |
|  | 48 | A113 | 0.0399666944213156 | 39.97 | 39.97 | 118.19 |
|  | 134 | A114 | 0.111573688592839 | 111.57 | 111.57 | 329.91 |
|  | 779 | G115  C116 | 0.648626144879267 | 648.63 | 89.04  571.19 | 263.29  1689.01 |
|  | 28 | A117 | 0.0233139050791007 | 23.31 | 23.31 | 68.93 |
|  | 2 | A118 | 0.00166527893422148 | 1.67 | 1.67 | 4.94 |
| Σ | 1201 |  | 1 | 1000 | 1000.01 | 2957.04 |

(4713 + 1201)/2 = 2957


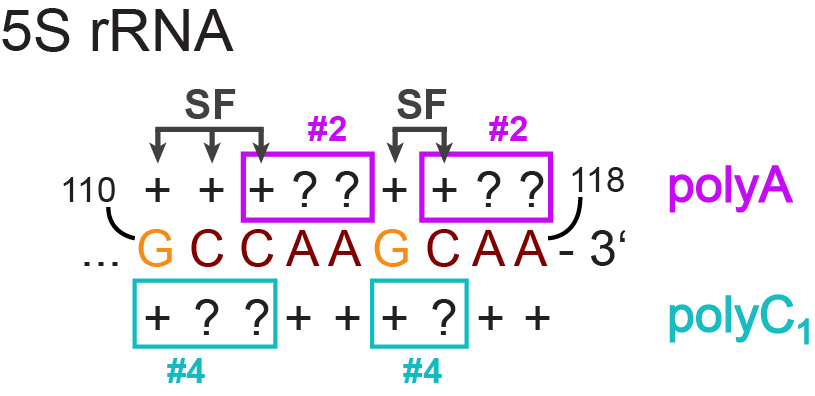


**Calculation of merged values using a pocket calculator**

Position A118/A117/C116 case #2

- totalA at C116/A117/A118: *f_1000_* = 606.2
- A118 unambiguous in polyC_1_: *f_1000_* = 1.67
- A117 unambiguous in polyC_1_: *f_1000_* = 23.31
- calculate *f_1000_* for C116 in polyA based on polyC_1_: 606.2 – 23.31 – 1.67 = 581.22

Position C116/G115 case #4

- G115 unambiguous in polyA: *f_1000_* = 90.6
- totalC at C116/G115: *f_1000_* = 648.63
- totalA at C116/G115: *f_1000_* =90.6 + 581.22 = 671.82
- define scaling factor (SF) for C116/G115 in polyA: *f_1000_* = 648.63/ (90.6 + 581.22) = 0.9655
- correct value for C116 in polyA with scaling factor (SF): *f_1000_* = 581.22 × 0.9655 = 561.16
- correct value for G115 in polyA with scaling factor (SF): *f_1000_* = 90.6 × 0.9655 = 87.47
- calculate merged mean value for C116: *f_1000_* = (581.22 + 561.16)/2 = 571.19 ± 14.18
- calculate merged mean value for G115: *f_1000_* = (90.6 + 87.47)/2 = 89.04 ± 2.21

Position A114/A113/C112 case #2

- A114 unambiguous in polyC_1_: *f_1000_* = 111.57
- A113 unambiguous in polyC_1_: *f_1000_* = 39.97
- calculate for C112 in polyA: *f_1000_* = 240.61 – 111.57 – 39.97 = 89.07

Position C112/C111/G110 case #4

- totalC at C112/C111/G110: *f_1000_* = 174.85
- totalA at C112/C111/G110: *f_1000_* = 89.07 + 42.22 + 20.37 = 151.66
- define SF for C112/C111/G110 in polyA: *f_1000_* = 174.85/(89.07 + 42.22 + 20.37) = 174.85/151.66 = 1.15291
- correct value for C112 in polyA with scaling factor (SF): *f_1000_* = 89.07 × 1.15291 = 102.69
- correct value for C111 in polyA with scaling factor (SF): *f_1000_* = 42.22 × 1.15291 = 48.68
- correct value for G110 in polyA with scaling factor (SF): *f_1000_* = 20.37 × 1.15291 = 23.48
- calculate merged mean value for C112: *f_1000_* = (89.07 + 102.69)/2 = 95.88 ± 9.63
- calculate merged mean value for C111: *f_1000_* = (42.22 + 48.68)/2 = 45.45 ± 4.57
- calculate merged mean value for G110: *f_1000_* = (20.37 + 23.48)/2 = 21.93 ± 2.

The values for for 'merged mean *f_1000_*' are finally divided by 1000 and are multiplied with the mean of the sum of read counts from both libraries (4713 + 1201)/2 = 2957 (see right column in the tables above).

**Supplementary Tables tRNA-Glu. Calculations underlying Fig. S7A**

**Merging of experimental polyA and polyC1 libraries for tRNAGlu (UUC)**

experimental polyA library tRNAGlu (UUC)

|  | read  counts (input) | position | fraction of  reads (*f*) | fraction  × 1000 (*f_1000_*)  (rounded) | merged *f_1000_* | merged read counts (output) |
| --- | --- | --- | --- | --- | --- | --- |
|  | 60 | U65 | 0.00495949743759299 | 4.96 | 2.48 | 16.73 |
|  | 0 | A66 |  | 0 | 0 | 0 |
|  | 101 | C67 | 0.00834848735328153 | 8.35 | 6.68 | 45.07 |
|  | 420 | G68 | 0.0347164820631509 | 34.72 | 19.15 | 129.21 |
|  | 79 | G69 | 0.00653000495949744 | 6.53 | 4.7 | 31.71 |
|  | 806 | G70 | 0.0666225822449992 | 66.62 | 59.46 | 401.18 |
|  | 592 | U71 | 0.0489337080509175 | 48.93 | 79.23 | 534.56 |
|  | 914 | C72  A73 | 0.0755496776326666 | 75.55 | 122.34  0 | 825.43  0 |
|  | 2217 | C74 | 0.183253430319061 | 183.25 | 134.88 | 910.04 |
|  | 6909 | C75  A76 | 0.571086129938833 | 571.09 | 313.21  257.88 | 2113.23  1739.92 |
| Σ | 12098 |  | 1 | 1000 | 1000.01 | 6747.08 |

experimental polyC1 library tRNA^Glu^ (UUC)

|  | read counts  (input) | position | fraction of  reads | fraction  × 1000 (*f_1000_*)  (rounded) | merged *f_1000_* | merged read counts (output) |
| --- | --- | --- | --- | --- | --- | --- |
|  | 0 | U65 | 0 | 0 | 2.48 | 16.73 |
|  | 7 | A66  C67 | 0.00501432664756447 | 5.01 | 0  6.68 | 0  45.07 |
|  | 5 | G68 | 0.00358166189111748 | 3.58 | 19.15 | 129.21 |
|  | 4 | G69 | 0.00286532951289398 | 2.87 | 4.7 | 31.71 |
|  | 73 | G70 | 0.0522922636103152 | 52.29 | 59.46 | 401.18 |
|  | 389 | U71  C72 | 0.27865329512894 | 278.65 | 79.23  122.34 | 534.56  825.43 |
|  | 558 | A73  C74  C75 | 0.399713467048711 | 399.71 | 0  134.88  313.21 | 0  910.04  2113.23 |
|  | 360 | A76 | 0.257879656160458 | 257.88 | 257.88 | 1739.92 |
| Σ | 1396 |  | 1 | 999.99 | 1000.01 | 6747.08 |

(1396 +12098)/2 = 6747

**
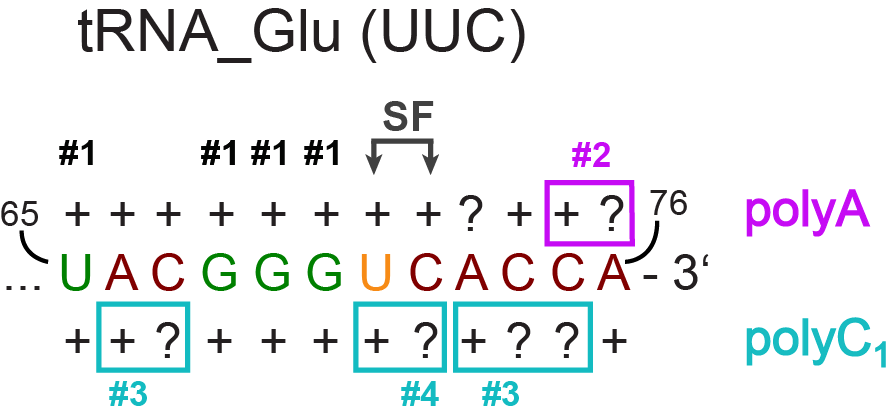
 Calculation of merged values using a pocket calculator**

Position A76/C75 case #2

- A76 unambiguous in polyC1: *f_1000_* = 257.88
- total at C75/A76 in polyA: *f_1000_* = 571.09
- calculate C75 in polyA: *f_1000_* = 571.09 – 257.88 = 313.21

Position C75/C74/A73 case #3

- C74 unambiguous case in polyA: *f_1000_* = 183.25
- total at A73/C74/C75 in polyC1: *f_1000_* = 399.71
- C75 in polyA calculated in previous step: *f_1000_* = 313.21
- calculate C74 in polyC1: *f_1000_* = 399.71 – 313.21 = 86.5
- 86.5 < 183.25 for C74 in polyA, thus no normalized read counts left for A73 🡪 A73 = 0
- calculate merged mean value for C74 = (183.25 + 86.5)/2 = 134.88 ± 68.41

Positions C72/U71 case #4

- *f_1000_* = 0 for A73 from previous step
- correct C72 in polyA: *f_1000_* = 75.55 – 0 = 75.55
- U71 unambiguous in polyA: *f_1000_* = 48.93
- totalC at U71/C72: *f_1000_* = 278.65
- totalA at U71/C72: 48.93 + 75.55 = 124.48
- calculate value for C72 in polyC1 with scaling factor (SF): *f_1000_* = 75.55 × 278.65/124.48 = 169.12
- calculate value for U71 in polyC1 with SF: *f_1000_* = 48.93 × 278.65/124.48 = 109.53
- calculate merged mean value for C72: *f_1000_* = (169.12 + 75.55)/2 = 122.34 ± 66.16
- calculate merged mean value for U71: *f_1000_* = (48.93 + 109.53)/2 = 79.23 ± 42.85

Position G70 case #1

- calculate mean value in polyA and polyC1 for G70: *f_1000_* = (66.62 + 52.29)/2 = 59.46 ± 10.13

Position G69 case #1

- calculate mean value in polyA and polyC1 for G69: *f_1000_* = (6.53 + 2.87)/2 = 4.7 ± 2.59

Position G68 case #1

- calculate mean value in polyA and polyC1 for G68: *f_1000_* = (34.72 + 3.58)/2 = 19.15 ± 22.02

Position C67/A66 case #3

- C67 unambiguous in polyA: *f_1000_* = 8.35
- totalC at A66/C67: *f_1000_* = 5.01
- negative value for A66 because totalC at A66/C67 is less than the totalA at C67: 5.01 – 8.35 = - 3.34 🡪 0 counts left for A66, *f_1000_* = 0 for A66.
- calculate mean value in polyA and polyC1 for C67: *f_1000_* = (5.01 + 8.35)/2 = 6.68 ± 2.36

Position U65 case #1

- calculate mean value in polyA and polyC1 for U65: *f_1000_* = (4.96 + 0)/2 = 2.48 ± 3.51

The values for for 'merged mean *f_1000_*' are finally divided by 1000 and are multiplied with the mean of the sum of read counts from both libraries, (1396 +12098)/2 = 6747 (see right column in the tables above)

**Supplementary Tables tRNA-Glu simulated. Calculations underlying Fig. S7B**

**Simulation of polyA/polyC libraries based on the experimental 3’-adapter library for tRNAGlu (UUC)**

experimental 3’-adapter library tRNA**Glu** (UUC)

|  | read  counts (input) | position | fraction of  reads (*f*) | fraction  × 1000 (*f_1000_*)  (rounded) |
| --- | --- | --- | --- | --- |
|  | 0 | U65 | 0 | 0 |
|  | 0 | A66 | 0 | 0 |
|  | 1 | C67 | 0.00471698 | 4.72 |
|  | 0 | G68 | 0 | 0 |
|  | 1 | G69 | 0.00471698 | 4.72 |
|  | 16 | G70 | 0.07547169 | 75.47 |
|  | 1 | U71 | 0.00471698 | 4.72 |
|  | 38 | C72 | 0.17924528 | 179.25 |
|  | 0 | A73 | 0 | 0 |
|  | 1 | C74 | 0.00471698 | 4.72 |
|  | 21 | C75 | 0.09905660 | 99.06 |
|  | 133 | A76 | 0.62735849 | 627.36 |
| Σ | 212 |  | 1 | 1000.02 |

**Simulation of polyA library for tRNA^Glu^ (UUC):**

Define the scaling factor (SF) for the simulated polyA library: **SF** = 12038/212 (sum of reads in polyA library without U65 divided by sum of reads in 3’-adapter library) = 56.783

- C75/A76 ambiguous in polyA: 21 + 133 reads in 3’-adapter library: 154 × **SF** = 8744.58
- C74 unambiguous in poly A: 1 read in 3’-adapter library: 1 × **SF** = 56.783
- C72/A73 ambiguous in polyA: 38 + 0 reads in 3’-adapter library: 38 × **SF** = 2157.75
- U71 unambiguous in polyA: 1 read in 3’-adapter library: 1 × **SF** = 56.783
- G70 unambiguous in polyA: 16 reads in 3’-adapter library: 16 × **SF** = 908.53
- G69 unambiguous in polyA: 1 read in 3’-adapter library: 1 × **SF** = 56.783
- G68 unambiguous in polyA: 0 reads in 3’-adapter library: 0 × **SF** = 0
- G67 unambiguous in polyA: 1 read in 3’-adapter library: 1 × **SF** = 56.783

simulated polyA library tRNAGlu (UUC)

|  | read counts  (rounded)  (input) | position | fraction of  reads | fraction  × 1000 (*f_1000_*)  (rounded) | merged *f_1000_* | merged read counts  (output) |
| --- | --- | --- | --- | --- | --- | --- |
|  | 0 | U65  A66 | 0 | 0 | 0  0.14 | 0  0.94 |
|  | 57 | C67 | 0.004734219 | 4.73 | 4.73 | 31.78 |
|  | 0 | G68 | 0 | 0 | 0 | 0 |
|  | 57 | G69 | 0.004734219 | 4.73 | 4.87 | 32.72 |
|  | 909 | G70 | 0.07549833887 | 75.5 | 75.33 | 506.1 |
|  | 57 | U71 | 0.004734219 | 4.73 | 4.73 | 31.78 |
|  | 2158 | C72  A73 | 0.17923588 | 179.24 | 179.24  0 | 1204.22  0 |
|  | 57 | C74 | 0.004734219 | 4.73 | 4.63 | 31.11 |
|  | 8745 | C75  A76 | 0.72632890365 | 726.33 | 99.27  627.06 | 666.95  4212.9 |
| Σ | 12040 |  | 1 | 999.99 | 1000 | 6718.5 |

**Simulation of polyC1 library for tRNAGlu (UUC):**

**­­­**Define the scaling factor (SF) for the simulated polyC library: **SF** = 1396/212 (sum of reads in polyC library divided by sum of reads in 3’-adapter library) = 6.585

- A76 unambiguous in polyC: 133 reads in 3’-adapter library: 133 × **SF** = 875.792
- A73/C74/C75 ambiguous in polyC: 0 + 1 + 21 reads in 3’-adapter library: 22 × **SF** = 144.868
- U71/C72 ambiguous in polyC: 1 + 38 reads in 3’-adapter library: 39 × **SF** = 256.811
- G70 unambiguous in polyC: 16 reads in 3’-adapter library: 16 × **SF** = 105.358
- G69 unambiguous in polyC: 1 read in 3’-adapter library: 1 × **SF** = 6.585
- G68 unambiguous in polyC: 0 reads in 3’-adapter library: 0 × **SF** = 0
- A66/G67 unambiguous in polyC: 0 + 1 read in 3’-adapter library: 1 × **SF** = 6.585

simulated polyC1 library tRNAGlu (UUC)

|  | read counts  (rounded)  (input) | position | fraction of  reads | fraction  × 1000  (rounded) | merged *f_1000_* | merged read counts (output) |
| --- | --- | --- | --- | --- | --- | --- |
|  | 0 | U65 | 0 | 0 | 0 | 0 |
|  | 7 | A66  C67 | 0.005010737 | 5.01 | 0.14  4.73 | 0.94  31.78 |
|  | 0 | G68 | 0 | 0 | 0 | 0 |
|  | 7 | G69 | 0.005010737 | 5.01 | 4.87 | 32.72 |
|  | 105 | G70 | 0.0751610594 | 75.16 | 75.33 | 506.1 |
|  | 257 | U71  C72 | 0.18396564066 | 183.97 | 4.73  179.24 | 31.78  1204.22 |
|  | 145 | A73  C74  C75 | 0.10379384395 | 103.79 | 0  4.63  99.27 | 0  31.11  666.95 |
|  | 876 | A76 | 0.627057981389 | 627.06 | 627.06 | 4212.9 |
| Σ | 1397 |  | 1 | 1000 | 1000 | 6718.5 |

**Merging of simulated polyA and polyC1 libraries for tRNAGlu (UUC)**

**Calculation of merged values using a pocket calculator**

**
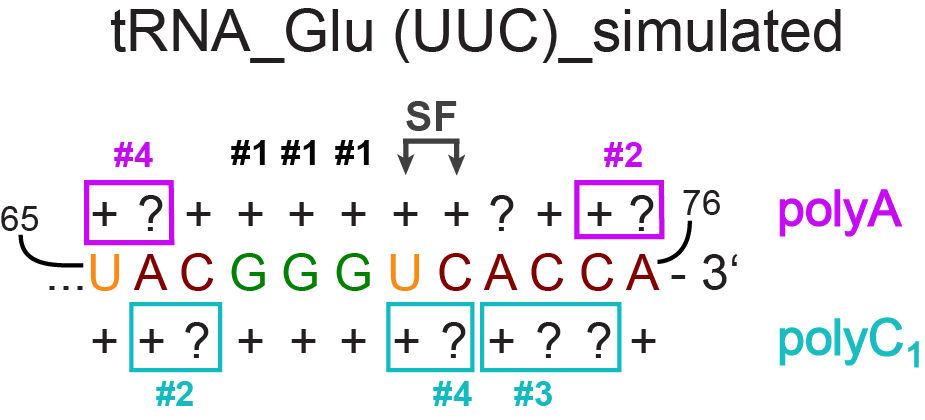
**

Position A76/C75 case #2

- A76 unambiguous in polyC1: *f_1000_* = 627.06
- total at C75/A76 in polyA: *f_1000_* = 726.33
- calculate C75 in polyA: *f_1000_* = 726.33 – 627.06 = 99.27

Position C74/A73 case #3

- C74 unambiguous case in polyA: *f_1000_* = 4.73
- total at A73/C74/C75 in polyC1: *f_1000_* = 103.79
- C74 in polyA calculated in previous step: *f_1000_* = 99.27
- calculate C74 in polyC: *f_1000_* = 103.79 – 99.27 = 4.52
- 4.52 < 4.73 for C74 in polyA, thus no normalized read counts left for A73 🡪 A73 = 0
- calculate merged mean value for C74 = (4.52 + 4.73)/2 = 4.63 ± 0.15

Positions C72/U71 case #4

- *f_1000_* = 0 for A73 from previous step
- correct C72 in polyA: = 179.24 – 0 = 179.24
- U71 unambiguous in polyA: *f_1000_* = 4.73
- totalC at U71/C72 in polyC1: *f_1000_* = 183.97
- C72 counts in polyA from previous step: *f_1000_* = 179.24
- totalA at U71/C72: 4.73 + 179.24 = 183.97
- calculate value for C72 in polyC1 with scaling factor (SF): *f_1000_* = 179.24 × 183.97/183.97 = 179.24
- calculate value for U71 in polyC1 with SF: *f_1000_* = 4.73 × 183.97/183.97 = 4.73
- calculate merged mean value for C72: *f_1000_* = (179.24 + 179.24)/2 = 179.24
- calculate merged mean value for U71: *f_1000_* = (4.73 + 4.73)/2 = 4.73

Position G70 case #1

- calculate mean value in polyA and polyC1 for G70: *f_1000_* = (75.5 + 75.16)/2 = 75.33 ± 0.24

Position G69 case #1

- calculate mean value in polyA and polyC1 for G69: *f_1000_* = = (4.73 + 5.01)/2 = 4.87 ± 0.2

Position G68 case #1

- *f_1000_* = 0 in both libraries

Position C67/A66 case #2

- C67 unambiguous in polyA: *f_1000_* = 4.73
- totalC at A66/C67: *f_1000_* = 5.01
- correct A66 in polyC1: *f_1000_* = 5.01 – 4.73 = 0.28
- calculate mean value for A66 in polyA and polyC1: *f_1000_* = (0.28 + 0)/2 = 0.14 ± 0.2

Position A66/U65 case #4

- A66/U65 unambiguous in polyA: *f_1000_* = 0
- U65 unambiguous in polyA: *f_1000_* = 0
- calculate result at position U65: *f_1000_* = (0 + 0)/2 = 0

The values for for 'merged mean *f_1000_*' are finally divided by 1000 and are multiplied with the mean of the sum of read counts from both libraries, (1397 +12040)/2 = 6718.5 (right columns in tables for simulated polyA and polyC_1_ libraries of tRNAGlu (UUC)).

**Supplementary References**

Redko Y, Bechhofer DH, Condon C. 2008. Mini-III, an unusual member of the RNase III family of enzymes, catalyses 23S ribosomal RNA maturation in *B. subtilis*. Mol Microbiol. 68:1096-1106.

Sogin ML, Pace NR. 1974. *In vitro* maturation of precursors of 5S ribosomal RNA from *Bacillus subtilis*. Nature. 252:598-600.

Suzuma S, Asari S, Bunai K, Yoshino K, Ando Y, Kakeshita H, Fujita M, Nakamura K, Yamane K. 2002. Identification and characterization of novel small RNAs in the *aspS-yrvM* intergenic region of the *Bacillus subtilis* genome. Microbiology (Reading) 148:2591-2598.

Tamaki S, Tomita M, Suzuki H, Kanai A. 2018. Systematic Analysis of the Binding Surfaces between tRNAs and Their Respective Aminoacyl tRNA Synthetase Based on Structural and Evolutionary Data. Front Genet. 8:227.

Wiegard JC, Damm K, Lechner M, Thölken C, Ngo S, Putzer H, Hartmann RK. 2023. Processing and decay of 6S-1 and 6S-2 RNAs in *Bacillus subtilis*. RNA 29:1481-1499.
